# Supplementary material for: Ratiometric detection of perfluoroalkyl carboxylic acids using dual fluorescent nanoparticles and a miniaturised microfluidic platform
Source: Nat Commun. 2025 Dec 2;16:10869. doi: 10.1038/s41467-025-66872-9 (PMC12675511; doi:10.1038/s41467-025-66872-9)
Supplement: Supplementary file 1 — Supplementary Information [file 41467_2025_66872_MOESM1_ESM.pdf]

Supplementary Information

Ratiometric detection of perfluoroalkyl carboxylic acids using dual fluorescent nanoparticles and a miniaturised microfluidic platform

Yijuan Sun,† Víctor Pérez-Padilla,† Virginia Valderrey, Jérémy Bell, Kornelia Gawlitza and Knut Rurack

Bundesanstalt für Materialforschung und -prüfung (BAM), Richard-Willstätter-Str. 11, 12489 Berlin, Germany.

E-mail: knut.rurack@bam.de

I. General experimental information S3
a. Chemicals and materials S3
b. Instrumentation and methods S3
c. Titration protocols S6
i. Monophasic titration protocol for indicator monomer **1** using cuvettes S6
ii. Protocol for assessment of complex stability under pre-polymerisation conditions S6
iii. Mono- and biphasic titration protocol for particle probes using cuvettes S7
d. Uncertainty budget[3] S7
e. Limit of detection[5] S8
f. Determination of binding constants (K_S) and apparent binding constants (K_S^{app}) S8
g. Evaluation of titration data with **gMIP@oSNP** S9
II. Synthesis and characterisation of indicator monomer **1** and model compound **4** S10
a. Synthesis of 2-(3-(4-(2,6-diethyl-1,3,5,7-tetramethyl-4,4-difluoro-4-bora-3a,4a-diaza-(s)-indacenyl)-phenyl)-thioureido)-ethyl methacrylate (**2**).[7] S10
b. Synthesis of 2,6-diethyl-1,3,5,7-tetramethyl-8-(4-(N,N-dimethylamino)phenyl)-4,4-difluoro-4-bora-3a,4a-diaza-(s)-indacene (**4**). S10
c. NMR spectra of indicator monomer **1**. S11
d. HRMS spectrum of indicator monomer **1** S13
III. Spectroscopic properties of indicator monomer **1**. S14
IV. Theoretical considerations on the signalling mechanism of indicator monomer **1** S15
V. Steady-state spectroscopic host-guest studies of indicator monomer **1** with PFOA S20

VI. Time-resolved fluorometric host-guest studies of indicator monomer **1** with PFOA 22
VII. Considerations on titrations of model systems 24
VIII. ^{1}H NMR host-guest studies of indicator monomer **1** 28
IX. Sensory particle preparation 33
a. Functionalisation of **oSNPs** with APTES (**a@oSNPs**) 33
b. Functionalisation of **a@oSNPs** with RAFT agent CPCTP (**raft@oSNPs**) 33
c. Preparation of orange silica nanoparticle core/green molecularly imprinted polymer shell particle probes (**gMIP@oSNP**) 33
X. Characterisation of particles 34
XI. Assays with **gMIP@oSNP** particle probes 39
XII. Microfluidics 45
a. Microfluidic platform 45
b. Mixing efficiency of the passive mixer 46
c. Microfluidic MIPs titration protocol 47
XIII. Comparison of selected fluorescence-based detection methods for PFAA from literature 48
XIV. References 51

I. General experimental information

a. Chemicals and materials

All reagents and solvents were obtained from commercial suppliers and used without further purification, except where indicated. Ammonia solution (32%) was purchased from AppliChem, 2,2′-azobis(2,4-dimethylvaleronitrile) (ABDV, >98.0%) from Wako, perfluorooctanoic acid (PFOA, 98%), 2-(perfluorooctyl)ethyl methacrylate (PFOEM, 97%), 2,4-dimethyl-3-ethylpyrrole (97%) and ammonium acetate (98%) from J&K, 1,1′-thiocarbonyldiimidazole, *N*,*N′*-dicyclohexylcarbodiimide (DCC), ammonia solution (7N in methanol), (3-aminopropyl)triethoxysilane (APTES, 99%), ethylene glycol dimethacrylate (EGDMA, 98%), ethyl chloroformate (EC, ≥98%), sodium dodecylbenzenesulphonate (SDBS), amoxicillin (AMOX, 90%), boron trifluoride diethyl etherate ($BF_3 \bullet Et_2O$), perchloric acid (70%, trace metal basis), rhodamine 6G (R6G, 99%) and aluminium oxide-based inhibitor removers from Sigma-Aldrich. 2-Aminoethyl methacrylate hydrochloride was obtained from Acros Organics, Triton X-100, sodium perfluorooctanoate (PFOA-Na, 97%), octanoic acid (OA, 98%), enoxacin (ENOX), *N,N*-diisopropylethylamine (DIEA, 99%) and ultrapure water from Alfa Aesar, tetraethyl orthosilicate (TEOS, >99%) and trifluoroacetic acid (TFA, 99%) from Honeywell, 4-cyano-4-(phenylcarbonothioylthio)pentanoic acid (CPCTP, >97%), perfluorooctanesulphonic acid (PFOS, 97%) and tris(2,2′-bipyridyl)ruthenium(II) chloride hexahydrate (98%) from abcr, triethylamine (TEA, ≥99.0%, ChemSolute), acetic acid (AcOH, 99%), sodium chloride (99%, ChemSolute), *n*-hexane (95%, ChemSolute) and hydrochloric acid (37%, ChemSolute) from Th. Geyer as well as sodium dodecyl sulphate (SDS) and sodium sulfate (99.99%) from Merck.

The solvents used for the synthesis were used as received from the supplier. Dichloromethane (CH_2Cl_2, ≥99.99%, ChemSolute), acetonitrile (MeCN, ≥99.9%), acetone (≥99.0%), anhydrous tetrahydrofuran (THF, ≥99.9%), cyclohexane (≥99.8%), ethanol 96% (EtOH, ≥95.1%), EtOH absolute (≥99.9%) and ethyl acetate (EtOAc, ≥99.5%) were purchased from Th. Geyer (all ChemSolute grade), anhydrous chloroform ($CHCl_3$) from Acros Organics, anhydrous toluene (99.8%) and *n*-hexanol (99%) from Alfa Aesar.

The solvents used for spectroscopic titrations were used as received from the supplier. MeCN, $CHCl_3$, EtOAc, THF, hexane, methanol (MeOH) and EtOH were purchased from Merck (all Uvasol grade), propyl acetate (PrOAc), also from Merck, was Analytical Grade (99%). Milli-Q water was obtained by running demineralised water (by ion exchange) through a Milli-Q ultrapure water purification system (Millipore Synthesis A10). Solvents were filtered with 0.2 µm Teflon filters prior to use in microfluidic experiments. Potential interferences by adsorption or desorption of fluorinated species to or from the tubing was found negligible, yielding recovery rates of 100±1 % for PFOA-spiked samples and no increasing signal for blank measurements, respectively.

b. Instrumentation and methods

Absorption and fluorescence spectroscopy, mass spectrometry (MS), nuclear magnetic resonance spectroscopy (NMR), thermogravimetric analysis (TGA), transmission electron microscopy (TEM), N_2 adsorption-desorption measurements and dynamic light scattering (DLS) techniques were employed to characterise the synthesised molecules and materials and to test their behaviour towards the corresponding analytes.

^{1}H and ^{13}C-NMR spectra were recorded with a Varian Mercury 400 NMR spectrometer. Samples were dissolved in CD_3CN or $CDCl_3$ (both from Sigma-Aldrich), using residual proton signals as internal standards (^{1}H: δ [CD_3CN] = 1.94 ppm, δ [$CDCl_3$] = 7.26 ppm and ^{13}C: δ[CD_3CN] = 1.32 and 118.26 ppm, δ[$CDCl_3$] = 77.16 ppm). Chemical shifts are represented in δ (ppm). Ultra-high-performance liquid chromatography electro-spray ionisation mass spectrometry (UPLC-ESI-MS) was performed on a Waters Acquity UPLC (gradient mixtures of acetonitrile (MeCN)/water) with a Waters LCT Premier XE mass detector. Additionally, a Waters Alliance System with

Waters Separations Module 2695, a Waters Diode Array Detector 996 and a Waters Mass Detector ZQ 2000 were used. Chromatographic separations were performed with a gradient of MeCN in water with 0.1% formic acid.

Absorption spectra and spectrophotometric titrations were acquired with a Specord 210 Plus spectrophotometer (Analytik Jena), using dilute solutions with an absorbance of around 0.1 at the absorption maximum. The molar absorption coefficients of **1** were determined from three independent double experiments, the value of ε_{452} = 14,500 M^{-1} cm^{-1} for $Ru(bpy)_3Cl_2$ was taken from ref. 1. Host–guest interaction was assessed via binding constants according to Equation S4 (below), being derived on the basis of a formalism for strong stoichiometric complex formation,[2] as well as via dose–response fitting according to Equations S5 and S6 (below) in case of analyte or binding site heterology. Fluorescence spectra and titrations were carried out on a FluoroMax 4 spectrofluorometer (Horiba Jobin-Yvon). Fluorescence lifetimes were determined with a customized time-resolved laser setup consisting of a regenerative Ti:sapphire amplifier (Solstice Ace 100F10K HP, MKS Spectra Physics) and an optical parametric amplifier (TPR-Topas-F with TPR-NMW-UV1-F, MKS Spectra Physics) as excitation source as well as a spectrograph (Kymera 328i-A, Andor) and a streak camera setup (C1483-130, C13440-20CU, Hamamatsu) with electronics (C10647-10, C1097-05, Hamamatsu) as detection unit. Time ranges of 1, 2, 5, 10 and 20 ns were selected for recording the decays, 20 ns corresponding to a time division of 55.3 ps per channel and yielding typical instrumental response functions of 30–300 ps (full width at half maximum), and an uncertainty of measurement of ±3 to ±30 ps. The laser beam was attenuated with a double prism attenuator from LTB and typical excitation energies were in the nanowatt-to-microwatt range. The fluorescence lifetime profiles were analysed with the High-Performance Digital Temporal Analyzer (HPD-TA) software package including the TA-fit with the global deconvolution fitting module (Hamamatsu). Further details on data fitting are given in Section VI below.

Fluorescence quantum yields (Φ_f) of all BODIPY-involving species have been determined relative to rhodamine 6G as stated in Table S4,[3] with measurement uncertainties of ±5% (for Φ_f > 0.2), ±10% (for 0.2 > Φ_f > 0.02), ±20% (for 0.02 > Φ_f > 5 × 10^{-3}), and ±30% (for 5 × 10^{-3} > Φ_f).

TEM measurements were done with a FEI Talos F200S operating at 200 kV. Samples were prepared on carbon thin film-modified copper grids (200 mesh) by drying 10 µL of a 0.1% (w/v) dispersion in EtOH. ImageJ software (National Institute of Health, US)[4] was used for calculating the average diameter of the silica core particles and the shell thickness of the core-shell hybrids. TGA analyses were carried out on a STA7200 (Hitachi High-Tech Analytical Science) thermobalance, using in a first step an oxidising synthetic air atmosphere (80 mL min^{-1}) with a heating program consisting of a ramp of 10 °C min^{-1} from 25 °C to 1000 °C and in a second step an isotherm (synthetic air, 80 mL min^{-1}) at 1000 °C for 10 min. N_2 adsorption-desorption isotherms were recorded with a Micromeritics ASAP2010 automated sorption analyser. The samples were degassed for 3 h at 200 °C in a vacuum. The specific surface areas were calculated from the adsorption data in the low-pressure range using the Brunauer-Emmett-Teller (BET) model. DLS studies were conducted using a Malvern Zetasizer Nano ZS, the nanoparticles (NPs) were subjected to ultrasonication for 15 min prior to a short centrifuge pulse (2495 ×*g*) to remove NP aggregates. Elemental analysis (EA) was performed with an EuroEA EA3100 Elemental Analyser to determine the amount of sulphur of the functionalized particles. This is achieved through sample combustion at a high temperature, separation of the resultant gaseous species and thermal conductivity detection.

Liquid chromatography-tandem mass spectrometry (LC-MS/MS) validation experiments were carried out after dilution of solutions containing PFOA by taking aliquots of the solutions, diluting them with ultrapure water and MeCN as well as an internal standard solution by a factor of 5 in fluorine-free polypropylene LC vials. Chromatographic separation was achieved on an Agilent ZORBAX RRHD Eclipse Plus C18, 3mm× 100 mm, 1.8 µm column installed on an Agilent 1290 Infinity II UHPLC system consisting of the following modules: Agilent 1290 Infinity II High-Speed Pump (G7120A), Agilent 1290 Infinity II Multisampler with Multiwash Option

(G7167B) and Agilent 1290 Infinity II Multicolumn Thermostat (G7116B). A gradient elution was performed with 5 mM ammonium acetate in water (mobile phase A) and methanol (mobile phase B) at 0.4 mL min^{-1} with a total run time of 17 min (Table S1). To minimize background PFAS contamination, the Agilent PFC-Free HPLC Conversion Kit (part number 5004 0006) and a PFC delay column (part number 5062 8100) for delaying potential per- or polyfluorochemicals impurities from the mobile phases was installed on the UHPLC system. The injection volume was 10 µL and the thermostat was kept at 55 °C.

Detection was done with an Agilent Ultivo Triple Quadrupole Mass Spectrometer (G6465B). Mass transitions and collision energy (CE) are given in Table S2. Table S3 contains further MS parameters.

Table S1. LC gradient

Time [min]	A [%]	B [%]
0.0	85	15
1.0	85	15
1.5	45	55
5.5	30	70
7.0	20	80
12.0	0	100
14.0	0	100
14.5	85	15
17.0	85	15

Table S2. Mass transitions and collision energy (CE)

Analyte	Precursor ion	Product ion	CE [V]
PFOA	413	369	8
PFOA	413	169	20
PFOA	413	219	16
$^{13}C_8$-PFOA	421	376	8
$^{13}C_8$-PFOA	421	172	16

Table S3. MS parameters

Parameter	Value
Gas flow (L min^{-1})	13.0
Nebulizer (psi)	35.0
Sheath gas flow (L min^{-1})	11.0
Capillary voltage (V)	4000 (negative setpoint)
Nozzle voltage (V)	1500 (negative setpoint)
Gas temperature (°C)	300
Sheath gas temperature (°C)	250

c. *Titration protocols*

i. Monophasic titration protocol for indicator monomer **1** using cuvettes

The binding of an analyte to indicator monomer **1** was studied by spectroscopic titrations using two different preparation procedures.

Important notice: Low concentrations of PFOA must never be prepared in glass containers, i.e., neither stock solutions (e.g., 1 mM) for titration experiments nor more diluted solutions such as for Job plot experiments. Poly(propylene) or quartz containers are preferable to avoid adsorption of PFOA on the surface of the container walls. When working in organic solvents only, the quality of the solvent is critical, and reproducibility can vary considerably from batch to batch, even between freshly opened bottles.

First, a solution of **1** was prepared in the respective solvent and diluted in a 10 mm pathlength quartz cuvette to a final concentration of 1 µM. A stock solution of the analyte (relevant concentration, e.g., 10 µM for PFOA) was then prepared, also containing **1** at 1 µM to account for dilution. For these experiments, only Uvasol-grade solvents were used. The analyte stock solution was titrated into the indicator solution until saturation was reached.

Second, a solution of **1** was prepared in spectroscopic MeCN (Uvasol grade) and diluted in a 10 mm pathlength quartz cuvette with the same solvent to a final concentration of 1 µM (3 mL). A stock solution of the analyte (1 mM) in HPLC-grade MeCN (≥99.9%) was then prepared and titrated into the indicator solution until saturation was reached. Dilution of **1** was accounted for prior to analysis. The total amounts of PFOA in selected samples from the titration experiments (equiv. PFOA: 0, 0.1, 0.2, 0.3, 0.4, 0.6, 0.8, 1.0, 1.4, 3.4) were validated by LC-MS/MS measurements, see above. It should be noted that because of the necessary workup before LC-MS injection, this method can only validate the total amount of PFOA in the sample at the various titration steps. While the recovery rate was 99 ± 1 % for the 1 mM stock solution of PFOA, the recovery rates found in parallel to the titration, i.e., pipetting the same volumes of stock solution into the vials for dilution, standard addition and MS analysis and into the cuvettes for dilution, complexation and spectroscopic measurements (in the range 0.1–3.5 µM) were between 87 and 140%, which is most likely due to the entirely organic workflow involving quartz cuvettes for the latter.

For obtaining Job Plots at micromolar concentration, 10 µM stock solutions of **1** and PFOA were prepared in spectroscopic MeCN (Uvasol grade). Solutions (2 mL) of different molar ratios were prepared from the equimolar solutions of **1** and PFOA and the actual absorbance measured in a 10 mm pathlength quartz cuvette. For the corresponding Job Plots at mM concentrations, 2 mM stock solutions of **1** and PFOA were prepared in spectroscopic MeCN (Uvasol grade) and solutions (15 µL) of different molar ratios were prepared from the equimolar solutions of **1** and PFOA before measuring the actual absorbances in 100 µm pathlength circular quartz microcuvettes.

ii. Protocol for assessment of complex stability under pre-polymerisation conditions

The formation of the complex between PFOA and indicator monomer **1** was studied by both absorption and fluorescence to assess whether the complex remains stable in the presence of the reagents necessary to produce a MIP, i.e., cross-linker EGDMA, co-monomer PFOEM and carrier particles **raft@oSNP**. Several solutions were prepared at a concentration identical to the concentration used during MIP synthesis (Table S10): **1** (0.9 mM) in MeCN, **1** (0.9 mM) in the presence of PFOA (0.9 mM), PFOA + EGDMA (44 mM), PFOA + EGDMA + PFOEM (8 mM) and PFOA + EGDMA + PFOEM + **raft@oSNP** particles (6.5 g L^{-1}) in MeCN. Afterwards, 15 µL of one of the solutions was pipetted onto a circular quartz cell with 100 µm optical path length to record the absorption and fluorescence spectra of the various stages of the pre-polymerisation mixture. For fluorescence, the emission and excitation spectra (λ_{exc} = 480 nm, λ_{em} = 550 nm) were measured in front-face geometry.

iii. Mono- and biphasic titration protocol for particle probes using cuvettes

The binding of an analyte to the particle probes **gMIP@oSNP** was also studied by fluorescence titration in monophasic solvent systems. A stock suspension of **gMIP@oSNP** particles (1 g L^{-1}) was prepared in the desired solvent and 100 µL were added to 1.5 mL solvent in a 10 mm pathlength quartz cuvette to yield a final concentration of 62.5 mg L^{-1}. The analyte stock solution was then titrated into the **gMIP@oSNP** particle suspension until saturation was reached. After each addition, the cuvette was mixed manually (shaking) for 2 min prior to fluorescence measurement.

For the detection of an analyte from aqueous solutions, water samples (MilliQ or river water) were spiked with known concentrations of an analyte. The assay procedure then involved two steps, i.e., (i) a liquid-liquid extraction step achieving phase transfer of the analyte into an organic phase by manual mixing of a water sample (8 mL) with an organic phase (4 mL), consisting of either EtOAc or a 1:1-mixture of MeCN and EtOAc, in a 14 mL tube for 2 min. For the second step, either (ii.a) an aliquot (3 mL) of the organic phase of the EtOAc/H_2O 1/2 (v/v) biphasic system was mixed with an equal volume of MeCN prior to fluorescence titration or (ii.b) an aliquot (3 mL) of the organic phase of the ternary MeCN/EtOAc/H_2O 1/1/2 (v/v/v) biphasic solvent system was directly used for the titration. In all cases, the final concentration of **gMIP@oSNP** was 62.5 mg L^{-1}; measurements were carried out in 10 mm quartz cuvettes. The mixtures were shaken for 2 min prior to fluorescence measurement. This procedure was repeated for every single step of titration until signal saturation was reached. The use of a ternary biphasic solvent system or a simple biphasic system with subsequent addition of MeCN showed no difference in the measured fluorescence signal enhancement per concentration of analyte and the courses of titration.

d. Uncertainty budget[3]

The total relative errors u_{tot} of the measurements were calculated for each point according to Equation S1, taking into account the errors coming from the following steps:

Preparation: u_p, including

- Weighing on precision balance: u_w = 0.8% (weighing of two substances: n_w = 2) and
- Dissolving with Eppendorf Reference pipette: u_s = 0.2% (dissolving of two substances: $n_s \geq 2$) and
- Dilution of solution: u_d = 0.2% (dilution of two stock solutions: $n_d \geq 2$)

Measurement: u_m, including

- Cell length (± 0.02 mm): u_l = 0.04% and
- Fluorescence measurements (w. fluorometer): u_{FM} = 0.06% or
- Fluorescence measurements (w. portable spectrometer): u_{PS} = 1.2%

Repetition of experiments (n_r = 3):

- Experiments with indicator monomer and analyte solutions: 3% < u_r < 10% or
- Experiments with particle probe suspensions and analyte solutions, no data treatment, spectral range of indicator monomer I_f[530–625 nm] considered: $u_r \leq$ 30% or
- Experiments with particle suspensions and analyte solutions, reduced signals, $\Delta F/F_0$[530–625 nm]: 6% < u_r < 17% or
- Experiments with particle suspensions and analyte solutions, spectra deconvoluted into F_{BDP} and F_{RBP} and ratiometric signals S considered, see Eq. 5: u_r = 5% or
- Experiments with particle suspensions and analyte solutions, reduced ratiometric signals, $\Delta S/S_0$: u_r = 3%

Total:

- Successive iteration of steps *i* accounted by multiplication factor: $n_i \times u_i$

$$u_{tot} = \sum \sqrt{(n_i \times u_i)^2} \qquad \text{Eq. S1}$$

For plots of I_f vs. c_{analyte}: $u_{tot}(I_f) = \sum\sqrt{(u_r/\sqrt{n_r})^2 + u_p^2 + u_m^2}$ Eq. S1a

Besides intensities I_f, the quotients and reduced intensities were also used, simplifying them here to $F_x/F_0 = Q_x$ and $(F_x - F_0)/F_0 = R_x$.

For plots of F_x/F_0 vs. c_{analyte}: $u_{tot}(Q_x) = \frac{F_x}{F_0} \times \sqrt{(u_x)^2 + (u_0)^2}$ Eq. S1b

with $u_x = u_0 = \sqrt{(u_r/\sqrt{n_r})^2 + u_p^2}$ with the respective individual uncertainties, see above.

Because $(F_x - F_0)/F_0 = F_x/F_0 - 1$, $R_x = Q_x - 1$ and therefore $u_{tot}(R_x) \equiv u_{tot}(Q_x)$. Eq. S1c

For S, S_x/S_0 and $(S_x - S_0)/S_0$, only the repeat uncertainties need to be considered because no experiments are involved and otherwise the same equations apply as for Q_x and R_x.

e. Limit of detection[5]

$$\text{LOB} = \text{mean}_{\text{blank}} + 1.645(\text{SD}_{\text{blank}})$$ Eq. S2

$$\text{LOD} = \text{LOB} + 1.645(\text{SD}_{\text{lowest conc sample}})$$ Eq. S3

In the microfluidics experiments, the $\text{mean}_{\text{blank}}$ was the average signal of a blank solution measured for 2 min (approx. 240 data points), the SD_{blank} was the standard deviation of the respective blank signals and $\text{SD}_{\text{lowest conc sample}}$ was the standard deviation of the sample with the lowest concentration that could be differentiated from the baseline of the sigmoidal fitting of the sample, measured for 2 min. The quoted LOD in the text was then the average of three repeats of each titration.

f. Determination of binding constants (K_S) and apparent binding constants (K_S^{app})

As is described in the text and in detail in Section VII below, the 1:1 complex formation between guest and host is obscured by homoconjugation of the guest in neat MeCN (possibly also involving heteroconjugation with water or other trace species) so that only the strict control of titration conditions in MeCN allowed to obtain typical titration curves for 1:1 binding as shown in Figures 3d and S9. These data were analysed according to a 1:1 binding model for strong stoichiometric complex formation (Equation S4)[2]

$$Y = Y_0 + \frac{Y_{lim} - Y_0}{2c_1}\left\{c_1 + c_{PFAA} + \frac{1}{K_S} - \left[\left(c_1 + c_{PFAA} + \frac{1}{K_S}\right)^2 - 4c_1c_{PFAA}\right]^{\frac{1}{2}}\right\}$$ Eq. S4

with $Y = \Delta F/F_0$. As also explained in the passages on molecular complex formation, the titration curves obtained for MeCN, EtOAc and PrOAc under conventional experimental conditions had an unusually slow onset, leading to an S-shaped curve often encountered in bioanalytical assays with binders of a certain heterogeneity, only that the heterogeneity was caused by homoconjugation of the guest in the present case. Accordingly, to enable comparability of the titration data in the three solvents investigated in this study, the titration curves obtained according to the first protocol given above were fitted to a dose–response function (Equation S5), accounting best for analyte heterology of the system in organic solvents:

$$y = A_2 + \frac{(A_1 - A_2)}{1 + (x/x_0)^p}$$ Eq. S5

Here, A_1 and A_2 are the maximum and minimum response, x is the concentration, x_0 the concentration at which the response is 50% (corresponding to EC50) and p the slope. As all the mechanistic considerations and data

suggest that there is only one highly emissive species involved (see Sections VI, VII), the 1:1 complex between **1** and an acid, we can assume that the response observed through the modulation of the fluorescence of indicator monomer **1** is linearly proportional to analyte concentration and, hence, it can also be assumed that $x_0 \approx (K_S^{app})^{-1}$. The validity of this assumption was assessed by analysing the titration data for **1** and OA shown in Figure S8 below through fitting them to both models, Equations S4 and S5, yielding K_S = 2034 ± 49 M^{-1} via Equation S4 (r^2 = 0.9996) and K_S^{app} = 1712 ± 80 M^{-1} via Equation S5 (r^2 = 0.9999), agreeing considerably well. For a better comparability of the data, we only mention the K_S^{app} data in the text.

*g. Evaluation of titration data with **gMIP@oSNP***

As described in the text on *Sensing performance of **gMIP@oSNP** particles*, the titration curves suggested a bimodal dose–response behaviour, resulting from indicator monomers residing in close cavities and indicator monomers residing in open cavities closer to the surface of the MIP layer, the relative amount of which depend on MIP shell thickness and cross-linking degree, i.e., can change slightly from batch-to-batch. Additionally, as is expected for polymer networks, the cavities of both populations differ at the microscopic scale so that the response behaviour of the entire ensemble is best described by a bimodal logistic dose–response model. Fitting of the titration data was thus performed with Equation S6:

$$y = A_1 + (A_2 - A_1)\left[\frac{h}{1 + 10^{(LOGx_{01} - x)p_1}} + \frac{1 - h}{1 + 10^{(LOGx_{02} - x)p_2}}\right] \qquad \text{Eq. S6}$$

in which all the variables are defined as above and *h* is the weighting factor between the two responses.[6]

II. Synthesis and characterisation of indicator monomer 1 and model compound 4

*a. Synthesis of 2-(3-(4-(2,6-diethyl-1,3,5,7-tetramethyl-4,4-difluoro-4-bora-3a,4a-diaza-(s)-indacenyl)-phenyl)-thioureido)-ethyl methacrylate (**2**).*[7]

To a solution of 2,6-diethyl-1,3,5,7-tetramethyl-8-(4-aminophenyl)-4,4-difluoro-4-bora-3a,4a-diaza-(*s*)-indacene (395 mg, 1 mmol), i.e., *meso*-(4-aminophenyl)-BODIPY,[8] in CH_2Cl_2 (60 mL), 1,1′-thiocarbonyl–diimidazole (214 mg, 1.2 mmol) was added. The reaction mixture was stirred for 20 h at room temperature. Then the mixture was filtered through a pad of silica gel and concentrated in a vacuum. The resulting crude *meso*-(4-isothiocyanatophenyl)-BODIPY (**3**) was used for the next step without further purification, in which 2-aminoethyl methacrylate hydrochloride (234 mg, 1.41 mmol) and triethylamine (0.26 mL, 1.87 mmol) were added to a solution of crude **3** (410 mg, 0.94 mmol) in CH_2Cl_2 (70 mL) at room temperature. After stirring for ca. 85 h until complete consumption of the isothiocyanate (monitored by TLC), the solvent was evaporated under reduced pressure, and the residue was purified by column chromatography on silica gel using EtOAc/CH_2Cl_2 (1/10, v/v) as eluent to yield the polymerizable thiourea-BODIPY **2** as a red powder (478 mg, 90%). ^{1}H NMR (400 MHz, $CDCl_3$): δ (ppm) = 8.27 (s, 1H), 7.36 (d, *J* = 8.4 Hz, 2H), 7.33 (d, *J* = 8.5 Hz, 2H), 6.70 (s, 1H), 6.06 (s, 1H), 5.57 (s, 1H), 4.39 (t, *J* = 4.7 Hz, 2H), 4.02 (t, *J* = 4.7 Hz, 2H), 2.52 (s, 6H), 2.29 (q, *J* = 7.4 Hz, 4H), 1.89 (s, 3H), 1.30 (s, 6H), 0.97 (t, *J* = 7.4 Hz, 6H). ^{13}C NMR (101 MHz, $CDCl_3$): δ (ppm) = 180.77, 167.62, 154.35, 138.63, 138.07, 137.00, 135.88, 134.42, 133.20, 130.79, 130.41, 126.52, 124.70, 63.18, 45.55, 18.39, 18.36, 17.18, 14.70, 12.65, 12.02, 11.98. HRMS(ESI+): m/z calculated for $C_{30}H_{37}BF_2N_4NaO_2S$ $[M+Na]^+$: 589.2596, found: 589.2562.

*b. Synthesis of 2,6-diethyl-1,3,5,7-tetramethyl-8-(4-(N,N-dimethylamino)phenyl)-4,4-difluoro-4-bora-3a,4a-diaza-(s)-indacene (**4**).*

The synthesis of **4** has been adapted from ref. [9]. 298 mg (2 mmol) 4-dimethylaminobenzaldehyde was dissolved in dry CH_2Cl_2 (200 mL) under N_2 atmosphere and mixed with two equivalents of 2,4-dimethyl-3-ethylpyrrole (492 mg, 4 mmol). After addition of catalytic amounts of TFA (three drops) the reaction mixture was stirred at room temperature for 3 h. After complete consumption of the aldehyde as controlled by TLC, the reaction product was oxidized by the addition of a solution of 454 mg (2 mmol) DDQ in 30 mL N_2-saturated CH_2Cl_2. After additional stirring for 10 to 15 min, 3 mL of DIEA and 3 mL of BF_3•Et_2O were added and stirring was continued for 30 min in darkness. The reaction mixture was washed with H_2O (2 × 150 mL) and NaCl solution (1 × 100 mL), dried over Na_2SO_4 and evaporated. Purification was carried out by column chromatography on silica gel with $CHCl_3$ and recrystallization from $CHCl_3$ / hexane, yielding orange-reddish, metallic crystals (390 mg, 46 %). ^{1}H NMR (400 MHz, $CDCl_3$): δ (ppm) = 7.06 (2H, d, *J* = 8.79 Hz), 6.78 (2H, d, *J* = 8.79 Hz), 3.02 (6H, s), 2.52 (6H, s), 2.30 (4H, q, *J* = 7.82 Hz), 1.39 (6H, s), 0.98 (6H, t, *J* = 7.82 Hz); ^{13}C NMR (100 MHz, $CDCl_3$): δ (ppm) = 152.94, 150.58, 141.56, 138.58, 132.33, 131.51, 128.97, 123.09, 112.34, 40.37, 17.08, 14.64, 12.42, 11.91; MS (FAB) m/z %: 423 (M^+); $C_{25}H_{32}BF_2N_3$ [423.36]: calc. C 70.93, H 7.62, N 9.93, found C 70.80, H 7.51, N 9.92.

*c. NMR spectra of indicator monomer **1***

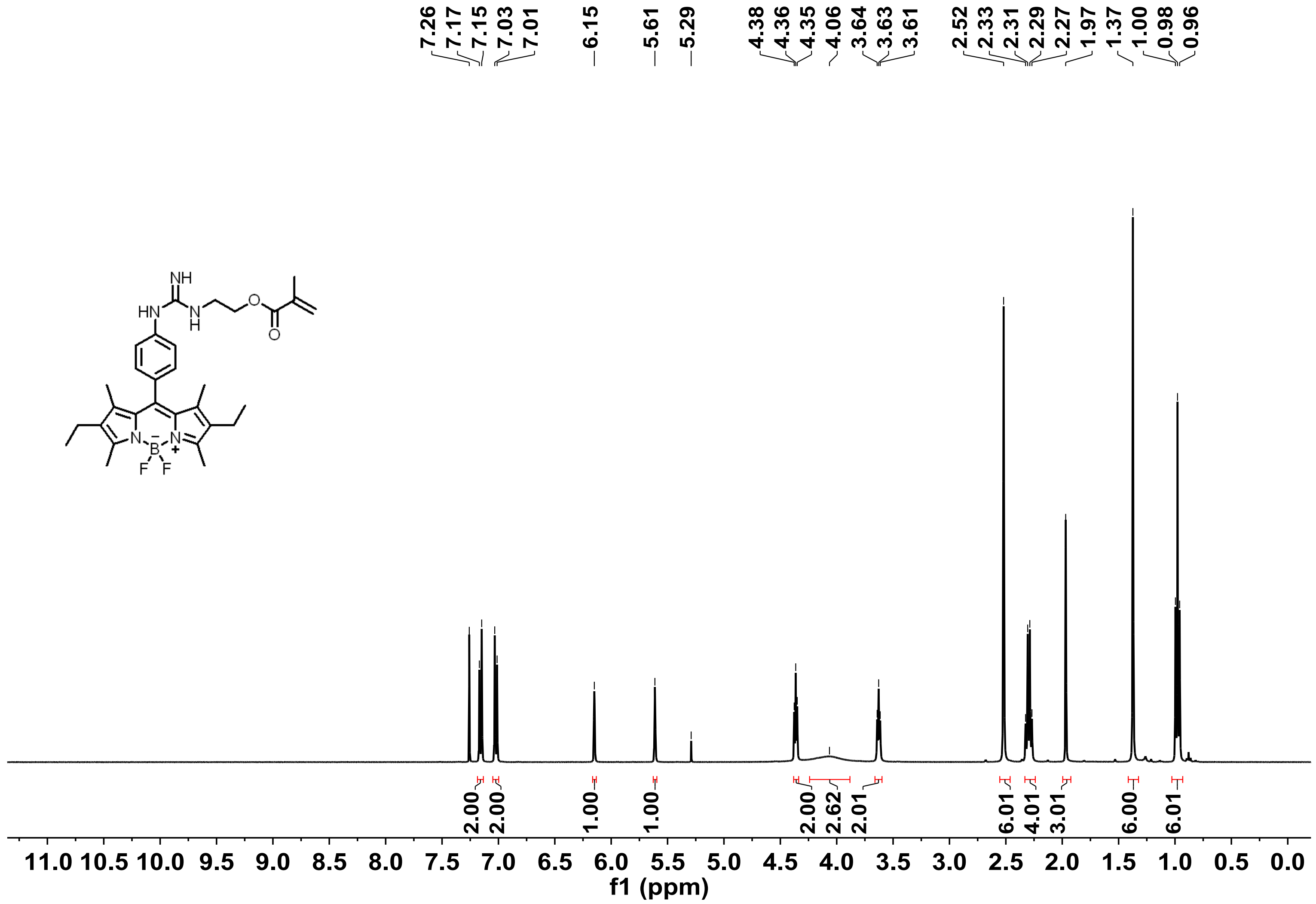

Figure S1. ^{1}H NMR (400 MHz) spectrum of guanidine-BODIPY **1** in $CDCl_3$ at 298 K.

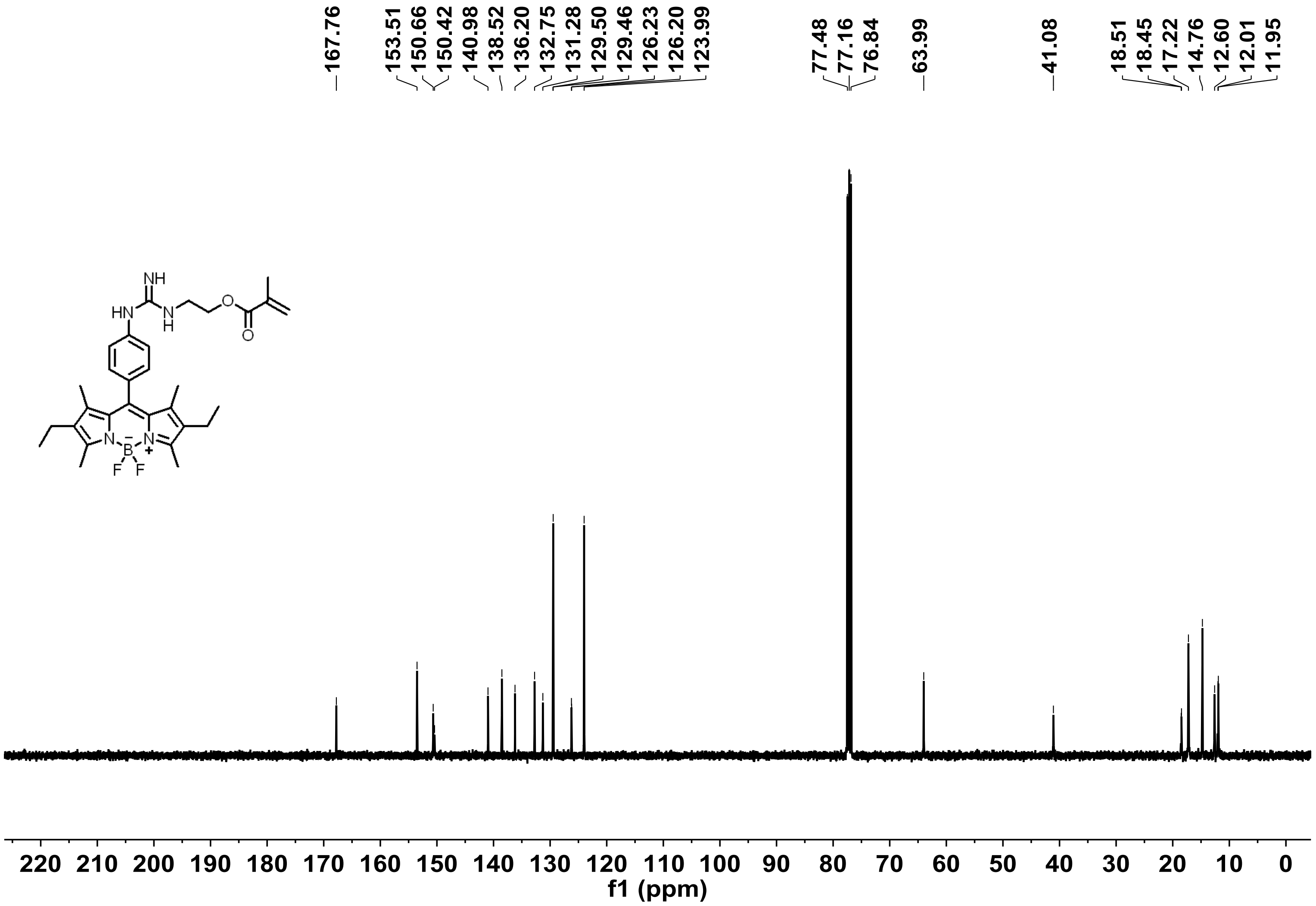

Figure S2. ^{13}C NMR (101 MHz) spectrum of guanidine-BODIPY **1** in $CDCl_3$ at 298 K.

*d. HRMS spectrum of indicator monomer **1***

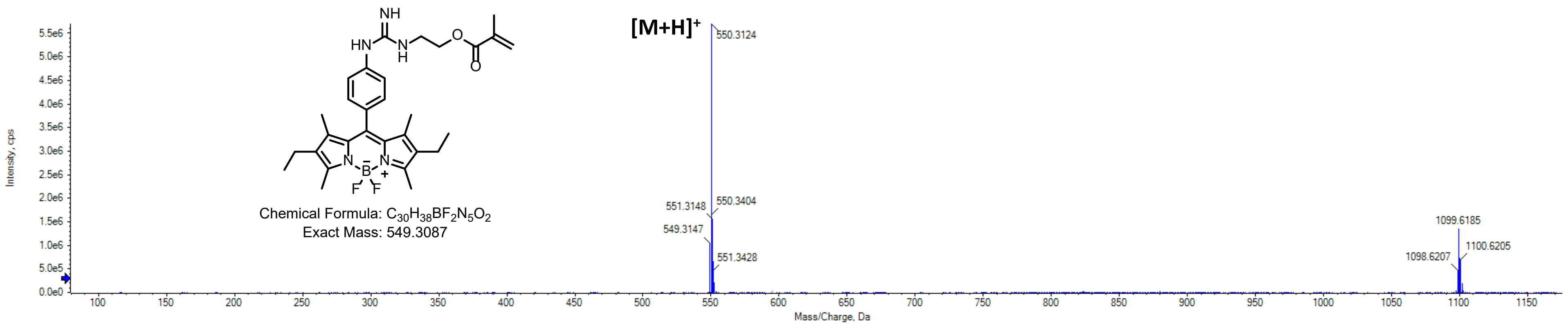

Figure S3. HRMS spectrum of guanidine-BODIPY **1**.

III. Spectroscopic properties of indicator monomer 1

Table S4. Spectroscopic properties of **1** in selected solvents at 298 K.

Solvent	$f_{\varepsilon,r}$[a]	λ_{abs} [nm]	λ_{em} [nm]	$\Delta\lambda_{abs-em}$ [nm]	Φ_f[b]
$CHCl_3$	0.254	527	537	10	0.58[c]
PrOAc[d]	0.283	521	532	11	0.04
EtOAc	0.292	521[e]	532	11	0.03
THF	0.308	523	534	11	0.02
EtOH	0.379	524	537	13	0.18[c]
MeCN	0.392	520[e]	531	11	0.01
MeOH	0.393	525	539	14	0.46[c]
MeCN/H_2O 20/80 v/v	0.402	524	537	13	0.71

[a] Solvent polarity function $f_{\varepsilon,r} = \frac{\varepsilon_r - 1}{2\varepsilon_r + 1} - \frac{1}{2}\frac{n^2-1}{2n^2+1}$, see discussion in ref [10]; ε_r and n for MeCN/H_2O mixture taken from[11, 12], for all other solvents from.[13] [b] Fluorescence quantum yield, determined relative to rhodamine 6G in ethanol (Φ_f = 0.91),[14] measurement uncertainty <0.05%. For a further classification of these data, see Section VI. [c] These values are apparent Φ_f obtained for conventional spectroscopic solvents used without prior treatment, i.e., which can contain traces of water or acid,[15] see text. [d] Propyl acetate. [e] Molar absorption coefficients, exemplarily determined to $\varepsilon = 76128 \pm 3113$ cm^{-1} M^{-1} and 70960 ± 1879 cm^{-1} M^{-1} in EtOAc and MeCN.

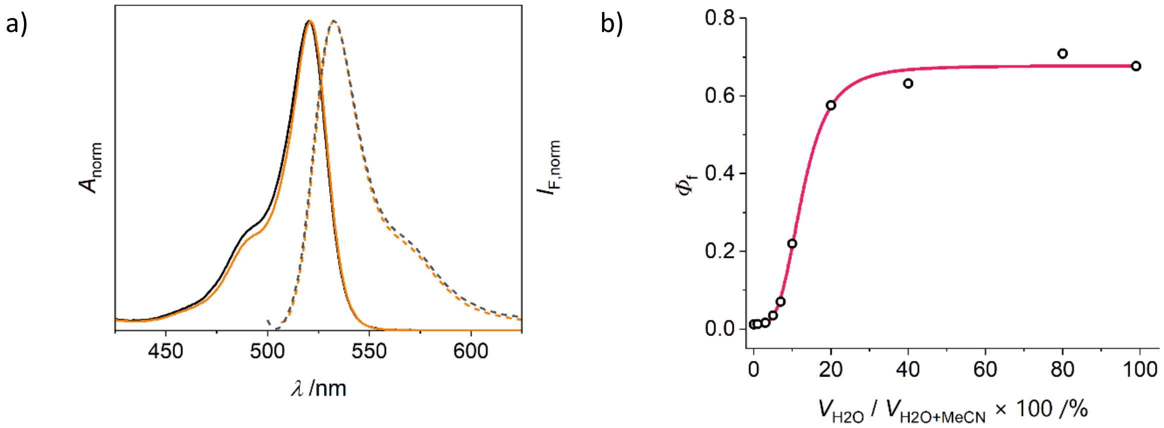

Figure S4. a) Normalised absorption (solid) and emission (dashed) of **1** in MeCN (black) and EtOAc (orange); b) Fluorescence quantum yield of **1** *vs* water content in MeCN/H_2O mixtures (λ_{exc} = 488 nm).

IV. Theoretical considerations on the signalling mechanism of indicator monomer 1

Following the discussion on which quantum chemical approaches can best reproduce the experimental absorption spectra and maxima for polymethine dyes, which include BODIPY dyes,[16, 17, 18, 19, 20, 21] we have chosen an approach that we recently identified as the optimal compromise between complexity and computing time for molecular systems like the one investigated here, i.e., systems for which the photophysical signalling behaviour is governed by both, polymethinic and charge/electron transfer characteristics.[7] Specifically, we employed density functional theory (DFT) and time-dependent density functional theory (TD-DFT) methods, using the B3LYP functional with the 6-31+G(d) basis set for geometry optimization and the CAM-B3LYP functional with the 6-311++G(d,p) basis set for vertical transitions.[7] In addition, the methacrylate group was neglected for **1** and trifluoroacetic acid (TFA), the simplest homologue of linear perfluorinated aliphatic carboxylic acids (PFCAs), was used instead of PFOA to reduce computation time. The quantum chemical calculations were performed and visualized with the software packages Gaussian 16 and GaussView 6[22, 23] and the relevant results are summarized in Tables S5–S7 and Figure S5, the essential ones are given in Figure 2a–c.

To recall, the experiments with **1**, PFOA and HCl revealed small bathochromic shifts in absorption upon interaction of **1** with the acids, accompanied by a significant enhancement of the fluorescence gradually increasing with solvent polarity as shown for **1** and PFOA in MeCN, EtOAc and PrOAc in Figures 3a,b and S7d. Accordingly, the fluorescence quantum yield of **1** (Φ_f = 0.01 in MeCN) is distinctly lower than for its *meso*-phenyl analogue (Φ_f = 0.87 in MeCN, see also the entry for **5** in Table S8 below),[24] suggesting that there is an active photophysical process significantly reducing the emission yield in **1** in highly polar solvents which can be switched back on again (Φ_f = 0.42 for **1**⊂PFOA in MeCN) in the desired signalling process through protonation-mediated binding of an acid.

If we inspect Table S5, containing the relevant theoretical results obtained on the three species **1**, **1**⊂TFA and **1**/HCl, it is evident that in all cases the lowest-energy transition is an oscillator-strong transition involving HOMO and LUMO. Figures 2a and S5 further show that both frontier MOs are localized on the BODIPY fragment (shortened to BDP in the tables) of the indicator. Accordingly, and as is typical for BODIPY-centred transitions, the dipole moment change between ground and excited state is small (Table S5, Figure 2b).[25] Whereas the second lowest transition is also BODIPY-localized for all three species, the S_3 transition involves a transition from the *p*-guanidino-phenyl (PhGua) fragment in the *meso*-position (HOMO−1) to the BODIPY core (LUMO) for **1** (Table S5, Figures 2a,b, S5). This transition is thus distinctly less oscillator-strong and accompanied by a large change in dipole moment of +18.6 D which is indicative of an excited-state charge (CT) or electron transfer (ET) process. Such processes have a forbidden character especially when the two subunits on which HOMO and LUMO are localized are orthogonally oriented, as in **1**, for which the optimized ground state geometry yields a dihedral angle of 83° between the BODIPY core and the phenyl substituent (see molecular structures as included in Figures 2a,b, S5).

For the two ion pairs **1**⊂TFA and **1**/HCl, transitions involving MOs localized on electronically largely decoupled molecular fragments of **1** are only found as S_4 transition at higher energies (Table S5, Figure 2b). However, these transitions have a reverse character, involving the BODIPY-localized HOMO and predominantly the PhGua-localized LUMO+2 for **1**⊂TFA as well as LUMO+1 for **1**/HCl (Table S5, Figures 2a,b, S5). This agrees well with the fact that the corresponding highest-energy PhGua-centred occupied MOs are found at distinctly lower energy for **1**⊂TFA (−8.44 eV, HOMO−3) and **1**/HCl (−8.89 eV, HOMO−6) than for **1** (−7.47, HOMO−1), see Figure S5. (It should be noted that both possible complex structures of **1**/HCl converge, i.e., Cl^- bound to the $=N^+H_2$ group as well as Cl^- bound to the Y-shaped motif. However, whereas the latter is even energetically slightly favoured, the dipole moment of the former is higher which would contribute to stabilization in polar solvents. As would be expected from

virtually decoupled indicators such as **1**, the calculated features of both complex conformers are very similar. For clarity, only the =N^+H_2 group bound **1**/HCl is discussed here.)

Table S5. Calculated properties of the vertical excitation of the most stable energy-minimized ground-state geometries of **1**, **1**⊂TFA and **1**/HCl as well as model compounds **4** and **4**/$HClO_4$ by TD-DFT; B3LYP functional with 6-31+G(d) basis set for geometry optimization and CAM-B3YLP functional with 6-311++G(d,p) basis set for vertical excitations.

	$\lambda_{S_n \leftarrow S_0}(n)$ /nm [a]	f [b]	$\Delta\mu_{S_n - S_0}$ /D [c]	fragment [d]	Orbitals (coefficients) [e]
**1**	435.4 (1)	0.592	−1.8	BDP	HOMO–LUMO (0.697)
	334.7 (2)	0.077	−0.5	BDP	HOMO−2–LUMO (0.696)
	323.3 (3)	0.038	+18.6	**PhGua–BDP**	HOMO−1–LUMO (0.682), HOMO−8–LUMO (−0.137)
	312.3 (4)	0.048	−0.7	BDP	HOMO−3–LUMO (0.694)
**1**⊂TFA	438.3 (1)	0.587	+0.1	BDP	HOMO–LUMO (0.697)
	336.7 (2)	0.076	−1.6	BDP	HOMO−1–LUMO (0.696)
	314.1 (3)	0.061	+1.9	BDP (PhGua–BDP)	HOMO−2–LUMO (0.679), HOMO−3–LUMO (0.151)
	280.6 (4)	0.003	+10.8	**BDP–PhGua**	HOMO–LUMO+2 (0.550), HOMO–LUMO+1 (−0.401)
**1**/HCl	440.0 (1)	0.586	0.0	BDP	HOMO–LUMO (0.697)
	337.5 (2)	0.076	−0.7	BDP	HOMO−4–LUMO (0.695)
	315.6 (3)	0.059	+0.8	BDP	HOMO−5–LUMO (0.694)
	290.7 (4)	0.004	+11.0	**BDP–PhGua**	HOMO–LUMO+1 (0.591), HOMO–LUMO+3 (−0.294), HOMO–LUMO+2 (−0.178)
**4**	436.7 (1)	0.589	−1.9	BDP	HOMO–LUMO (0.697)
	359.1 (2)	0.035	+20.0	**PhAn–BDP**	HOMO−1–LUMO (0.687), HOMO−6–LUMO (0.144)
	334.6 (3)	0.076	−0.5	BDP	HOMO−2–LUMO (0.696)
**4**/$HClO_4$	440.2 (1)	0.592	+0.8	BDP	HOMO–LUMO (0.697)
	337.6 (2)	0.078	+0.1	BDP	HOMO−1–LUMO (0.696)
	315.9 (3)	0.057	+0.4	BDP	HOMO−2–LUMO (0.695)
	299.5 (4)	0.003	+15.2	**BDP–Ph**	HOMO–LUMO+1 (0.679), HOMO–LUMO+2 (−0.155)

[*a*] Wavelength of the transition, calculated for a medium polar solvent such as chloroform according to a polarizable continuum model (IEFPCM). [*b*] Oscillator strength of the transition. [*c*] Dipole moment difference between ground (μ_0) and respective excited (μ_n) state. [*d*] Fragment on which the transition is mainly localized. [*e*] MOs involved in the transitions.

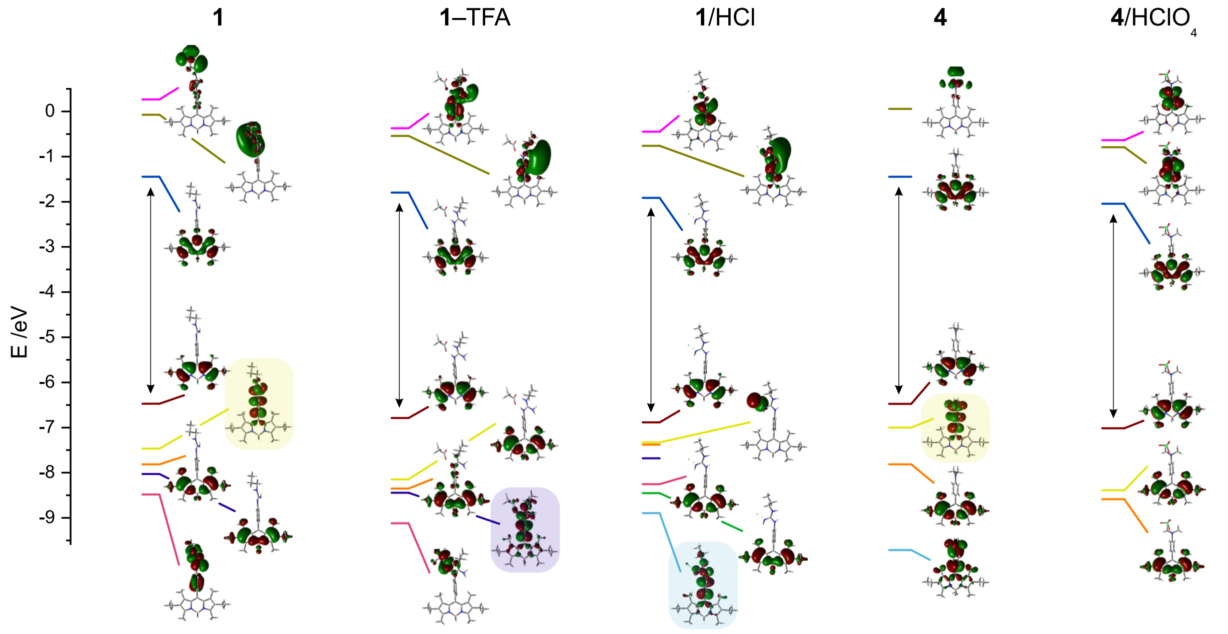

Figure S5. Energy levels of relevant frontier molecular orbitals HOMO (to HOMO−6) and LUMO (to LUMO+2) of **1**, **1**⊂TFA, **1**/HCl, **4** and **4**/$HClO_4$; localization of MOs on molecular fragments is shown for relevant MOs. Specific highlighting is explained in the text. B3LYP functional with 6-31+G(d) basis set for geometry optimization and CAM-B3YLP functional with 6-311++G(d,p) basis sets for vertical excitations. For **1**/HCl, HOMO−1, HOMO−2 and HOMO−3 are exclusively localized on the chloride ion and LUMO+3 is localized on the identical fragment as LUMO+2. For **4**, HOMO−3 to HOMO−5 are localized on BDP (−3, −5) or phenyl (−4) and are not shown for clarity.

The data included in Table S5 and Figure S5 for the model compound **4**, *meso*-(*p*-(*N*,*N*-dimethyl-amino)phenyl-2,6-diethyl-1,3,5,7-tetramethyl-BODIPY, and **4**/$HClO_4$ support the chosen design rationale. The features of the orbitals, transitions and states are qualitatively similar to **1**, only a stronger quenching is expected due to the quenching transition being S_2 and the energy gap between S_1 and S_2 being smaller, which has also been experimentally verified with a Φ_f = 0.003 in MeCN, see Table S8 below. Accordingly, and because **4** does not possess an anion receptor unit, also a stronger fluorescence enhancement is expected upon protonation, which has been verified experimentally, i.e., a 250-fold enhancement for **4**/$HClO_4$, see Table S8.

However, and although the data in Table S5 give first hints on different excited state deactivation pathways for **1** *vs* **1**⊂TFA and **1**/HCl as well as **4** and **4**/$HClO_4$, the potentially relevant quenching ET state in **1** is approx. 1 eV higher lying in energy than the emissive BODIPY-centred state in the gas phase (and still 0.6 eV higher lying in **4**). As such states are at the basis of fluorescent BODIPY indicators using basic aromatic units in the *meso*-position,[26, 27] the states were analysed in more detail with respect to the question if stabilization by solvents of different polarities can lead to a state reversal so that the quenching ET state becomes the energetically most favoured one (Figure 2c, Table S6).

Table S6. Calculated electronic properties of the BODIPY-localized (^{1}LE) and the ^{1}ET states of **1** using the results of the DFT/TD-DFT calculations shown in Table S5.

	S_n	solvent	$\Delta E_{S_n \leftarrow S_0}(sol)$ [a] /eV	$\Delta E_{S_n \leftarrow S_0}(gas)$ [b] /eV	$\Delta\mu_{S_n - S_0}$ [c] /D	μ_{S_n} [d] /D	f [e]	$\Delta E_{S_n - T_n}$ [f] /eV
^{1}LE	S_1	MeCN	2.89	2.85	−1.8	+7.5	0.592	1.66
^{1}ET	S_3	MeCN	**2.69**	3.83	+18.6	+27.8	0.038	0.00
^{1}LE	S_1	EtOAc	2.88	2.85	−1.8	+7.5	0.592	1.66
^{1}ET	S_3	EtOAc	**2.88**	3.83	+18.6	+27.8	0.038	0.00
^{1}LE	S_1	PrOAc	2.88	2.85	−1.8	+7.5	0.592	1.66
^{1}ET	S_3	PrOAc	**2.90**	3.83	+18.6	+27.8	0.038	0.00

[a] Calculated according to $\Delta E(sol) = \Delta E(gas) - \frac{1}{a_O{}^3}\frac{\epsilon_S - 1}{2\epsilon_S + 1}\left(\mu_{S_n}{}^2 - \mu_{S_n}\mu_{S_0}\right) - \frac{1}{a_O{}^3}\frac{n^2-1}{2n^2+1}\left(\mu_{S_n}\mu_{S_0} - \mu_{S_0}{}^2\right)$,[28, 29] with ϵ_S = 35.94, 6.02 and 5.62 for MeCN, EtOAc and PrOAc,[13, 30] n = 1.344, 1.372 and 1.383 for MeCN, EtOAc and PrOAc;[13, 30] a_O = 6.2 Å, determined according to molecular volumes[31] and corrected for non-spherical shape[32] for emissive species **1**; μ_{S_0} and μ_{S_n} obtained from DFT/TD-DFT calculations. [b] Energy of transition as obtained from TD-DFT calculations of the complexes in the gas phase, see Table S5. [c] Dipole moment difference between ground (μ_{S_0}) and respective excited (μ_{S_n}) state. [d] Dipole moment of excited state (μ_{S_n}). [e] Oscillator strength of the transition. [f] Energy gap between respective singlet and triplet states.

The results in Table S6 and Figure 2c indeed reveal that especially highly polar solvents should stabilize the ET state in **1** to such a degree that quenching occurs. For instance, whereas in the gas phase the energy gap for the ^{1}ET state is higher than that for the BODIPY-localized ^{1}LE state, $\Delta E_{S_n - S_0}$= 3.83 eV *vs* 2.85 eV, this sequence is reversed in MeCN (2.69 *vs* 2.89 eV). Although the theoretical analysis predicts this effect in absolute terms only for MeCN, the levelled states for EtOAc (both 2.88 eV) and the only slightly positive value for PrOAc (+0.02 eV) reflect well the trend of quenching, MeCN > EtOAc > PrOAc, and are attributed to the general differences between experimental and theoretical studies when considering, for instance, solvent-induced effects that are based on chemical interactions. Moreover, when the same analysis is done for **1**⊂TFA and **1**/HCl, this interpretation along with the revival of fluorescence upon binding of an acid becomes apparent (Table S7, Figure 2c).

The data in Table S7 and Figure 2c clearly reveal that the ET-triggered quenching is much less likely to occur after protonation of the guanidine receptor by an acidic guest, converting this unit into a positively charged guanidinium unit. Although the differences between the ^{1}LE and the ^{1}ET state are reduced for both **1**⊂TFA and **1**/HCl when considering a highly polar solvent such as MeCN (by ca. 0.4 eV, see Table S7), a state reversal is not found and the quenching ^{1}ET states are still >0.9 eV situated above the emissive ^{1}LE states. The additional data on **4** and **4**/$HClO_4$ included in Table S7, i.e., pronounced state reversal for **4** but none for **4**/$HClO_4$ stress the mechanistic design considerations and the fact that the photophysical signalling mechanism of **1** is well in line with the protonation of *meso*-aniline substituted BODIPY dyes, which are well-known fluorescent indicators for the acidic pH range.[27]

Table S7. Calculated electronic properties of the BODIPY-localized (^{1}LE) and the ^{1}ET states of **1**⊂TFA, **1**/HCl, **4** and **4**/$HClO_4$ in MeCN using the results of the DFT/TD-DFT calculations shown in Table S5.

	S_n	solvent	$\Delta E_{S_n \leftarrow S_0}(sol)$ [a] /eV	$\Delta E_{S_n \leftarrow S_0}(gas)$ [b] /eV	$\Delta\mu_{S_n - S_0}$ [c] /D	μ_{S_n} [d] /D	f [e]	$\Delta E_{S_n - T_n}$ [f] /eV
^{1}LE	S_1	**1**⊂TFA	**2.83**	2.83	+0.1	+10.8	0.587	1.69
^{1}ET	S_4	**1**⊂TFA	4.01	4.42	+10.8	+21.4	0.003	0.04
^{1}LE	S_1	**1**/HCl	**2.82**	2.82	0.0	+11.3	0.586	1.70
^{1}ET	S_4	**1**/HCl	3.74	4.27	+11.0	+22.3	0.004	0.04
^{1}LE	S_1	**4**	2.88	2.85	−1.9	+5.8	0.589	1.65
^{1}ET	S_2	**4**	**2.09**	3.45	+20.0	+27.7	0.035	0.25
^{1}LE	S_1	**4**/$HClO_4$	**2.79**	2.82	+0.8	+10.6	0.592	1.62
^{1}ET	S_4	**4**/$HClO_4$	3.33	4.14	+15.1	+24.9	0.003	0.04

[a] Calculated for MeCN as solvent according to $\Delta E(sol) = \Delta E(gas) - \frac{1}{a_O{}^3}\frac{\epsilon_S - 1}{2\epsilon_S + 1}\left({\mu_{S_n}}^2 - \mu_{S_n}\mu_{S_0}\right) - \frac{1}{a_O{}^3}\frac{n^2-1}{2n^2+1}\left(\mu_{S_n}\mu_{S_0} - {\mu_{S_0}}^2\right)$,[28, 29] with ϵ_S = 35.94 for MeCN,[30] n = 1.344 for MeCN;[30] a_O = 6.8 Å, 6.4 Å, 6.0 Å and 6.3 Å determined according to molecular volumes[31] and corrected for non-spherical shape[32] for species **1**⊂TFA, **1**/HCl, **4** and **4**/$HClO_4$; μ_{S_0} and μ_{S_n} obtained from DFT/TD-DFT calculations. [b] Energy of transition as obtained from TD-DFT calculations of the complexes in the gas phase, see Table S5. [c] Dipole moment difference between ground (μ_{S_0}) and respective excited (μ_{S_n}) state. [d] Dipole moment of excited state (μ_{S_n}). [e] Oscillator strength of the transition. [f] Energy gap between respective singlet and triplet states.

V. Steady-state spectroscopic host-guest studies of indicator monomer 1 with PFOA

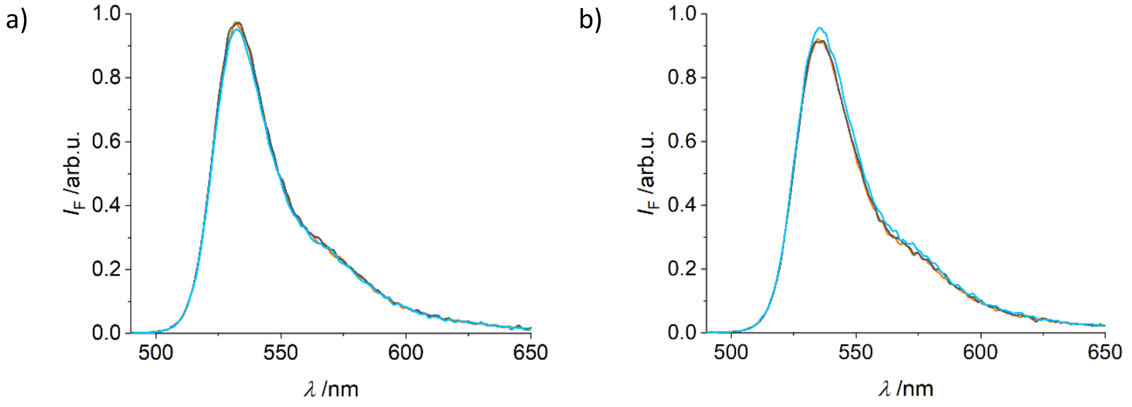

Figure S6. Emission of **1** in MeOH (a) and EtOH (b) in the absence (orange) and upon addition of PFOA at 50 (black) and 100 µM (blue); c_1 = 1 µM, λ_{exc} = 480 nm.

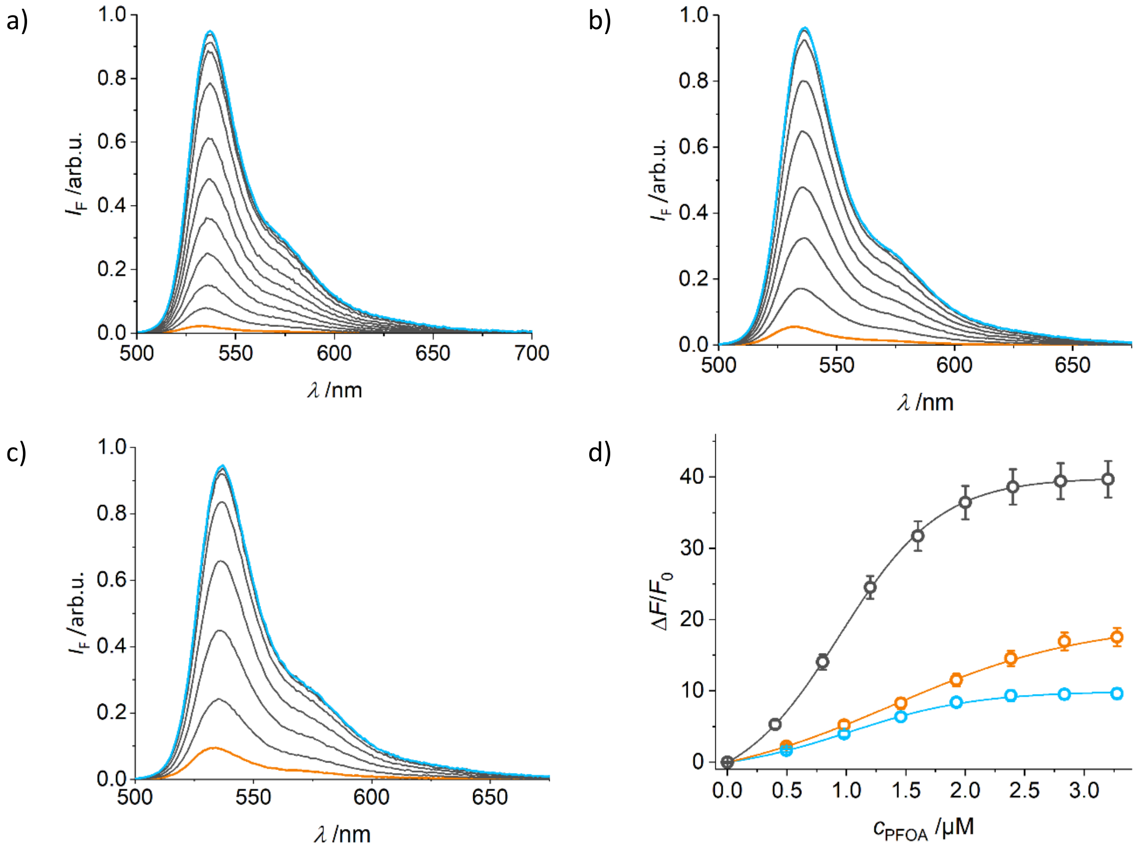

Figure S7. Emission of **1** in the absence (orange) and presence of increasing concentrations of PFOA in a) MeCN, b) EtOAc and c) PrOAc (end point spectra in blue) for titrations conducted according to the first protocol. d) Dose–response fitting (Equation S5) of the integrated emission intensities from a)–c) plotted in reduced fluorescence against PFOA concentration: MeCN (black), EtOAc (orange), PrOAc (blue); c_1 = 1 µM, λ_{exc} = 480 nm. For d), data are presented as measurement uncertainties, see Eq. S1b (n_r = 3 independent experiments).

Table S8. Spectroscopic properties of selected model compounds in MeCN at 298 K.

Compound	λ_{abs} /nm	λ_{em} /nm	$\Delta\lambda_{abs-em}$ /nm	Φ_f	τ_f /ns	*Ref.*
**4**	519	535	16	0.003[a]	<0.01	this work
**4**/$HClO_4$	525	541	15	0.75[a]	4.82	this work
**5**	522	533	11	0.87	5.25	[24]

[a] Fluorescence quantum yield, determined relative to *meso*-phenyl-1,3,5,7-tetramethyl-BODIPY in MeCN (Φ_f = 0.60),[26] measurement uncertainty <0.05%.

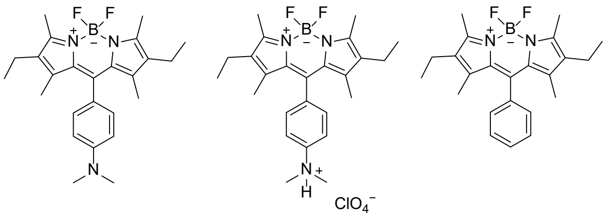

4 **4**/$HClO_4$ **5**

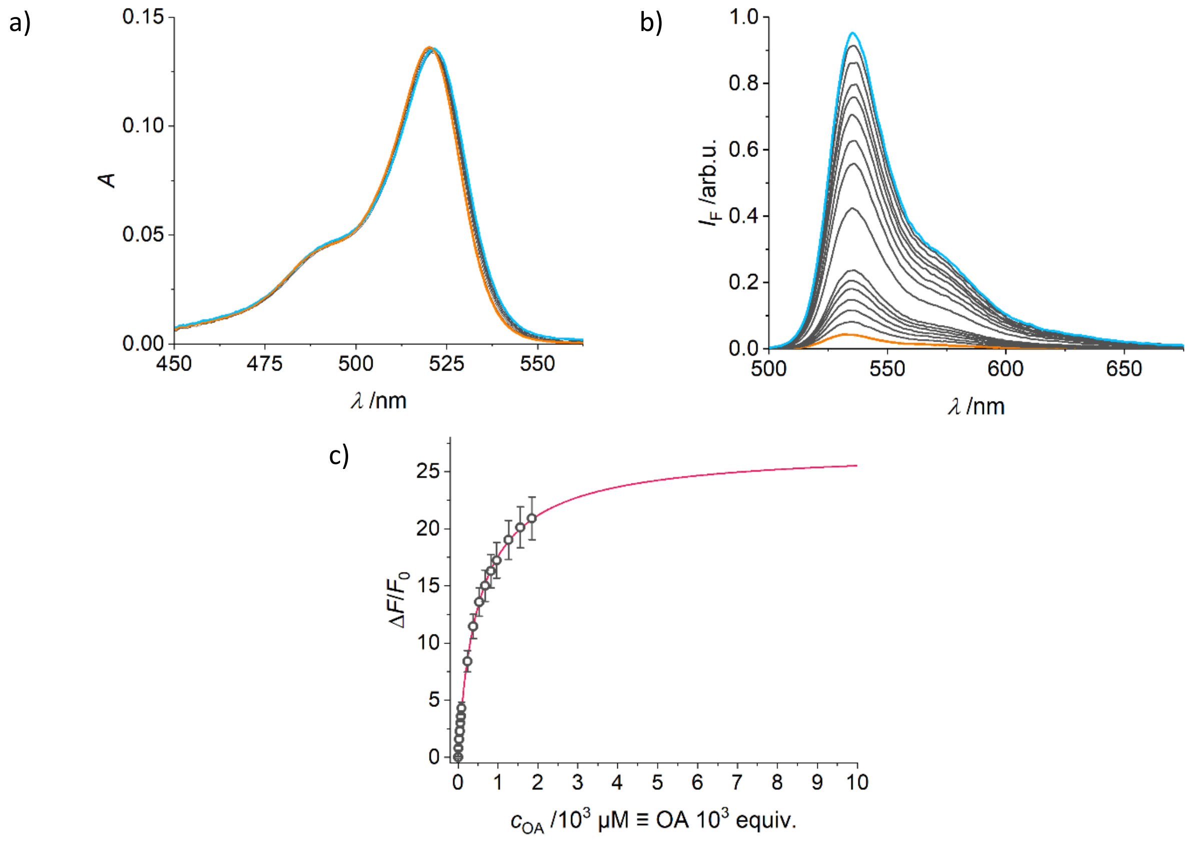

Figure S8. a) Absorption and b) emission of **1** in the absence (orange) and presence of increasing concentrations of octanoic acid (OA) from 0.01–1.85 mM in MeCN (end point spectra in blue) as well as c) corresponding response of **1** vs equivalents of octanoic acid (fit in red); c_1 = 1 µM, λ_{exc} = 480 nm. For c), data are presented as measurement uncertainties, see Eq. S1b (n_r = 3 independent experiments).

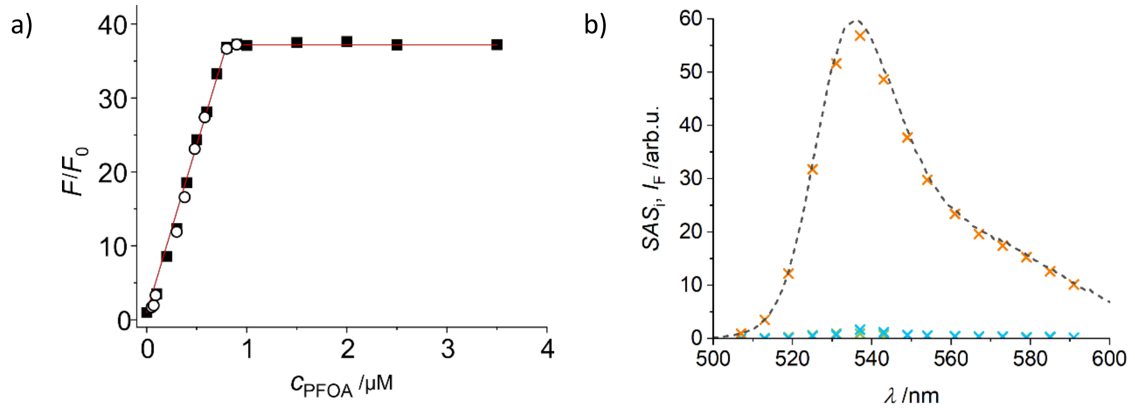

Figure S9. a) Steady-state fluorescence titration data of Figure 3d, including fit of the black data points according to a 1:1 stoichiometry (Equation S4), yielding $K_S > 10^7$ M^{-1}. b) Species associated spectra (SAS_i) of the three species (×, colour code as in Figure 3d: green for $i = 1$ [$\tau_1 = 0.03$ ns], blue for $i = 2$ [$\tau_2 = 0.51$ ns], orange for $i = 3$ [$\tau_3 = 4.35$ ns]); steady state spectrum included as dotted line for comparison) reconstructed from the wavelength-resolved fluorescence decays of **1** in the presence of 0.3 equiv. PFOA according to Equation S9; $c_1 = 1$ µM, $\lambda_{exc} = 480$ nm.

VI. Time-resolved fluorometric host-guest studies of indicator monomer 1 with PFOA

In addition to the experimental details reported in Section I.b., fitting of the fluorescence lifetime data had to be done in multiple stages. This was necessary, because the shortest and longest lifetime components found differed by more than two orders of magnitude, thus not allowing for the recording of an entire decay (20 ns time window) with a single time resolution that, at the same time, allows the binning of the photons of the fast component into a sufficient number of channels to determine the associated lifetime with good accuracy.

First, to select the best compromise between time range and resolution for the more in-depth analysis of the decays, a 10 nm-wide range around the emission maximum was selected from the three-dimensional camera images of samples showing predominantly the short (approx. 30 ps, free **1**) as well as the long (approx. 4.3 ns, saturated signal of **1**⊂PFOA) decay component and analysed according to decay models with one, two and/or three components. As a result, the 5 ns time range proved to yield the best possible results and most reliable and robust fits for the two components differing by more than two orders of magnitude. Second, the three-dimensional camera images of each decay of the various species measured were grouped into ten regions of 5 nm (between 510–610 nm) and fitted according to decay models with one, two and/or three components. This was followed by linking the lifetimes of these species and globally analysing the 5 nm decay traces until satisfactory fits were obtained, to assess whether the relative amplitudes of the decay components showed significant trends across the emission band being indicative of pronounced differences in decay associated spectra. As would be expected for systems like the present one where even protonation induces only small shifts of <5 nm, no significant spectral trends were found. Thus, third, for the fitting of the titration data, decay traces from 25 nm-broad regions (515–540 nm) were fitted individually to decay models with two and three components, revealing that the entire system **1**/PFOA under the present experimental conditions requires three decay components to yield satisfactory fits. In a final step, the 25 nm-decay traces of nine titration steps were fitted globally until a satisfactory result was obtained. The goodness-of-fit of the single decay regions and the global analysis were judged by reduced chi-squared (χ_R^2) and the autocorrelation function C(j) of the residuals. The best global fit, in which no parameter was fixed, resulted in decay times of 0.032, 0.512 and 4.350 ± 0.003 ns and a global χ_R^2 =

1.286. Using only two decay components, the best global fit resulted in a χ_R^2 = 2.332 and showed pronounced systematic deviations in the residuals and autocorrelation functions.

However, as is obvious especially from the top panel of Figure 3c, the time range of 5 ns does not allow to collect dark noise in channels after the fluorescence has fully decayed, so that the fitting programme is adjusting the background only through the evaluation of approx. 20 channels before the onset of the decay curve. Since this can lead to a certain misdetermination of the long lifetime, the decays of fully protonated **1**/HCl and fully complexed **1**⊂PFOA were also measured with a 20 ns time window, their analysis yielding the lifetimes included in Table S9.

Secondary data analysis included the reconstruction of the decay associated spectra (DAS) and the species associated spectra (SAS) from the results of the three component fits. Considering that the decay of a single component *i* is defined as in Equation S7

$$I_f^{tot}(\lambda, t) = \sum_i a_i(\lambda, \tau_i) e^{-t(\tau_i)^{-1}} \quad \text{Eq. S7,}$$

in the absence of an excited state reaction like here (strong complex formation in the ground state, absence of rise times), the DAS and SAS are given by Equations S8 and S9 as

$$DAS_i(\lambda, \tau_i) = \frac{n_i(\lambda) F^{SS}(\lambda)}{\sum_i n_i(\lambda)\tau_i} \quad \text{Eq. S8}$$

and

$$SAS_i(\lambda) = DAS_i(\lambda, \tau_i)\tau_i \quad \text{Eq. S9.}$$

Table S9. Fluorescence lifetime data and photophysical parameters of **1**, **1**⊂PFOA and **1**/HCl in MeCN at 298 K.

**Species**	τ_f **/ns**	Φ_f	k_r [a] **/10^8 s^{-1}**	k_{nr} [a] **/10^8 s^{-1}**
**1**	0.032 [b]	0.003 [c]	1 [d]	310 [d]
**1**⊂PFOA	4.16 [e]	0.42	1.0	1.4
**1**/HCl	4.23 [e]	0.52	1.2	1.1

[a] Radiative rate constants (k_r) and nonradiative rate constants (k_{nr}) were calculated using the following two equations: $k_r = \Phi_f/\tau_f$ and $k_{nr} = (1-\Phi_f)/\tau_f$. [b] Obtained from fits with high time-resolution. [c] Corrected steady-state value considering that the long decay component contributes with 2% to the decay in as-received spectroscopic MeCN employed for the measurements. [d] Because the lifetimes and relative amplitudes depend slightly on the time range and resolution chosen for measurements and fits, these data have a greater uncertainty than the data of **1**⊂PFOA and **1**/HCl. [e] Obtained from fits of 20 ns time range.

VII. Considerations on titrations of model systems

The shape of the titration curves of the indicator monomer **1** with PFOA in MeCN, EtOAc and PrOAc conducted under conventional experimental settings (first protocol, Section I.c.i.) as shown in Figure S7d is peculiar for the designed 1:1 stoichiometry of the recognition system, suggesting (an)other process(es) to be active. However, Figure S9a shows that this behaviour can approach the traditional 1:1 shape, the envisaged stoichiometry having also been found for the weaker binding OA (Figure S8). Moreover, the NMR titrations in Figure S16 also reveals the typical behaviour of 1:1 binding for **1** and both, the stronger (PFOA) and the weaker binding guest (OA), see also Figure S12 (blue circles) for PFOA. The apparent discrepancy can be explained with the fact that many acids, especially oxoacids, tend to undergo homoconjugation in organic solvents, this type of dimerization having an impact on the amount of available free anions for a given acid concentration and therefore also on the apparent pK_a^{app} of the acid in the non-aqueous environment (pK_a^{app} because the pK_a is defined in water).[33]

Homoconjugation means that the acid is not only existing as neutral and deprotonated species (Equation S10 = Equation 1 in text, acidity constant K_a^{app}), but that the acid also forms a dimeric species (Equation S11 = Equation 2, homoconjugation constant K_{AHA-}).

$$HA \rightleftharpoons A^- + H^+ \qquad \text{Eq. S10}$$

$$HA + A^- \rightleftharpoons [AHA]^- \qquad \text{Eq. S11}$$

Depending on K_a^{app} and K_{AHA-}, the number of free protons provided by two molecules of a certain acid under specific conditions can vary between two and one, in the latter case for the overall reaction (Equation S12).

$$2HA \rightleftharpoons [AHA]^- + H^+ \qquad \text{Eq. S12}$$

The situation is further complicated by the fact that not only homoconjugation can occur but that acids in organic solvents can also undergo heteroconjugation (Equation S13 = Equation 3), which in turn has an additional impact on the number of available free protons per molecule of acid, lying between one and zero in the extreme case.

$$HR + A^- \rightleftharpoons [AHR]^- \qquad \text{Eq. S13}$$

Here, HR is considered as an impurity in the system, and is in most experimental cases typically water which is still present in traces especially if solvents and compounds have not been meticulously dried and if the system has not been investigated under inert conditions. Heteroconjugation with water as HR can play a significant role if inorganic acids such as HCl are investigated in organic solvents.

If the heteroconjugation case is neglected for our present system, the homoconjugate and the free anion will certainly have distinctly different affinities for a protonated guanidinium binding site as in **1**, because $[AHA]^-$ will presumably possess a structure such as

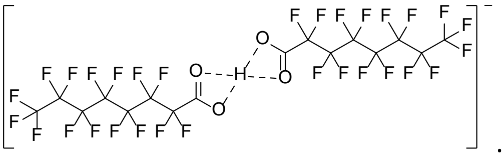

. Such structures, as well as neutral dimers, have recently been found in the gas phase by ion mobility spectrometry (IMS) and FTIR studies.[34, 35] $[AHA]^-$ is much bulkier than PFOA, the anionic subunit is much larger and its charge density much lower, and the ability to form directional hydrogen bonds with a complementary motif such as guanidinium is also much lower. Therefore, $[AHA]^-$ will not only have a different K_a^{app} for the gross equilibrium in Equation S12

than the K_a^{app} in Equation S10, but it will presumably also have a different—most likely lower—K_S for binding to **1**H^+, which in turn would reduce its tendency to protonate **1**.

The fact that strong complexes are formed, that the fluorescence lifetime measurements only suggest the presence of two major species, one weakly and one highly emissive, and that we validated the spectroscopic titrations with LC-MS measurements strongly suggests that a single strong and highly emissive complex is responsible for the spectroscopic response. In addition, Figures S10a,b show that the shifts of the absorption maxima correlate well with the fluorescence enhancement factors (FEF = F/F_0) derived from Figure S7a and that only the shift of the emission maximum is distinctly stronger. The latter is due to the explanation provided above for DAS and SAS, i.e., that the fluorescence quantum yields of the major species differ dramatically, leading to the pronounced dominance of SAS_3 in Figure S9b, i.e., that the emission shift is virtually completed at much smaller amounts of PFOA because Φ_f of **1**⊂PFOA is much larger than that of **1**. In addition, Figures S10c,d show that independent of the titration protocol used, sharp isosbestic points are found for the titration that yields a more S-shaped curve (Figure S10a) as well as for the titration that yields a more typical curve (Figure 3a).

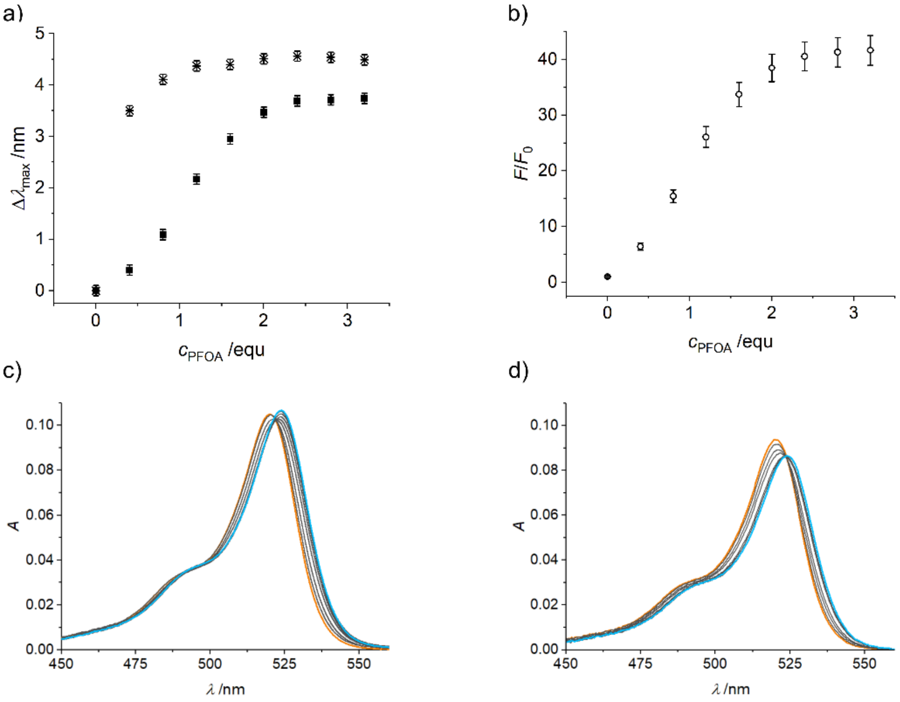

Figure S10. a) Shifts of absorption (squares) and emission (stars) maxima and b) fluorescence enhancement factor during the titration of **1** with PFOA shown in Figure S7a (top). Bottom: Absorption titration spectra according to c) the first titration protocol, corresponding to the data in Figures S7a,d and Figures S10a,b, and d) the second titration protocol, corresponding to the data in Figure 3a; all in MeCN. The maxima were derived by fitting the most intense sub-band of each spectrum to a lognormal function. For a), error bars were taken from the fit results. For b), data are presented as measurement uncertainties, see Eq. S1b (n_r = 3 independent experiments).

Because no rise times have been found in the fluorescence lifetime measurements, the presence of a specific excited-state deactivation pathway can also be ruled out (Figures 3c–e, S9b and Table S9). Whereas free **1** shows a fast decay of 32 ps, the lifetime of **1**⊂PFOA and **1**/HCl lie in the same range as that for highly emissive BODIPY dyes, i.e., approx. 4 ns. Moreover, when time-resolved fluorescence measurements are carried out according to the first titration protocol, leading to the S-shaped curves, a virtually identical decay behaviour is found, i.e., lifetimes of 0.036, 0.508 and 4.354 ns with the intermediate species remaining always below a_i^{rel} < 10%. Thus, the existence of two highly emissive complex species could not be established here and the chemical nature of the minor intermediate

component, showing τ_f ~0.5 ns and thus pointing to an only moderate Φ_f by assuming constant k_r for species involving the BODIPY core of **1** as the emitter, remains unknown at present. Because it shows the same concentration-dependent trend as the highly emissive species, see Figure 3d, it is highly unlikely that these species are **1**⊂PFOA and a putative species **1**/H[PFOA$_2$H], which presumably shows a much lower binding constant because of lower charge density and less electrostatic attraction, higher steric crowding, the unavailability of a hydrogen bonding motif and the overall absence of a dedicated binding mode for a guanidinium receptor. Moreover, two species always account for ≥90% of the spectroscopic signals. Despite these findings, the unusual shapes of the titration curves in Figure S7d, which cannot be fitted by 1:1, 2:1 or 1:2 complex stoichiometries in meaningful ways, remain.

As a straightforward way to assess the stoichiometry of a complex is a Job plot, for instance, carried out according to the method of continuous variations,[36] we conducted such a series of experiments to obtain better insight into the present system under conventional titration conditions (first protocol). Because of the large difference in fluorescence quantum yields between free and complexed **1**, which aggravate such experiments, UV/vis spectrophotometry was used for the corresponding experiments. The similarity of the trends between absorption spectral shifts and FEF, which can be seen in Figure S10, emphasizes the validity of this approach. Two experiments were carried out, with 10 µM and 2 mM solutions of host and guest so that at x = 0.5 the concentrations of $c_1 = c_{PFOA}$ = 5 µM and 1 mM. As can be seen in Figure S11, two different maxima were found for the two different concentrations. For the lower concentration, a maximum was found at a molar fraction of x_{PFOA} = 0.66, suggesting a complex stoichiometry of **1**⊂PFOA$_2$ (black data in Figure S11).[36, 37] In contrast, the 2 mM solutions yielded a maximum at a molar fraction close to 0.5 which corresponds to a 1:1 stoichiometry (red data in Figure S11). Since the higher concentrated solutions are closer to the conditions used for the NMR titrations, which were indicative of 1:1 binding, these results agree well.

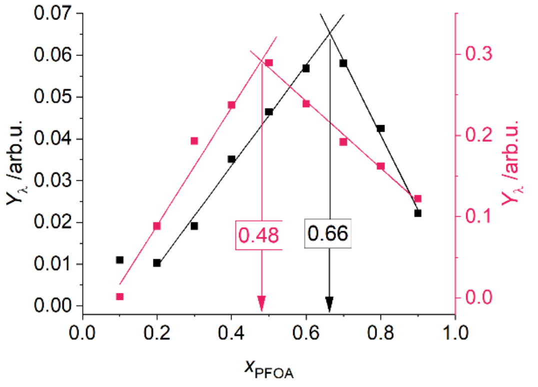

Figure S11. Job plots for **1** and PFOA at different concentrations in MeCN: 10 µM (black), 2 mM (red). $Y_\lambda = A_\lambda - A'_\lambda$ is the difference between the actually measured absorbance A_λ and the theoretically calculated absorbance assuming that no interaction occurs between the two species, $A'_\lambda = x_1 \varepsilon_{\lambda,1} c_1 d + x_{PFOA} \varepsilon_{\lambda,PFOA} c_{PFOA} d$ with x_n = molar fraction, $\varepsilon_{\lambda,n}$ = molar absorption coefficient and c_n = concentration of the species 1 and PFOA; d = optical path length. Fits indicated as straight lines and graphically obtained maxima as arrows.

However, besides these supposedly concentration-dependent stoichiometries, another aspect in Figure S11 deserves attention, i.e., that there appears to be no interaction between the two partners in the first two steps in the 10 µM series. Only at x_{PFOA} = 0.3 does Y_λ increase. This suggests that at low PFOA concentrations there is only a negligible amount of PFOA$^-$ present in solution and only [PFOA$_2$H]$^-$/H$^+$ (or perhaps even neutral PFOA$_2$ dimers?) exist, which do not interact with **1**. The deviation from the normal shape of a 1:1 titration curve in Figure S7d thus does not seem to be related to different complexes between **1** and PFOA$^-$ or different anionic species of PFOA, but to the species

heterogeneity of PFOA in MeCN as such and the availability of deprotonated, free PFOA⁻ as the only anionic species that binds strongly to **1** while generating a spectroscopic response.

In view of the findings of the Job plots, we went back to the spectroscopic titrations and conducted additional titrations at approx. twice the indicator concentration according to the first (conventional) protocol. In addition, we constructed an apparent titration curve from the Job experiments with 2 mM solutions, as the concentration of the indicator is distinctly higher than for the conventional titrations at every step in which indicator is involved. Figure S12 shows the $\Delta\lambda_{max}$ in absorption for these experiments. It is obvious that, starting from the titration of **1** at 1 µM with PFOA (black data), a progressive increase in the indicator concentration and thus in the PFOA concentration causes the S-shaped curve to gradually approach the shape of a typical 1:1 titration curve at upper micro- and lower millimolar concentrations (from black squares via green and hollow red squares to blue circles, Figure S12), which ultimately correspond to the curves obtained according to the second protocol (Figures 3c, S9a). All these experiments thus also point to an explanation that involves predominantly a single spectroscopically active complex species, **1**⊂PFOA, yet that the exact concentration of PFOA⁻ in the single experiments is unknown and depends on the concentration range investigated (and the quality of the solvent used). However, because of the following aspects

- no values for K_a^{app} and K_{AHA-} of PFOA in MeCN (as well as in EtOAc or PrOAc) could be found in the literature,
- already the pK_a of PFOA in water is a matter of debate, with values between −0.5 and 3.8 having been reported in the literature[38, 39, 40, 41, 42, 43] and
- except for neutral and protonated **1** all the other species escape optical detection,

we refrained from trying to disentangle the quantitative relationships within the scope of this work and described the binding behaviour by a nonlinear dose–response behaviour that can better account for analyte heterology as observed here. A more detailed mechanistic analysis with acids for which more tabulated data are available is currently under way.

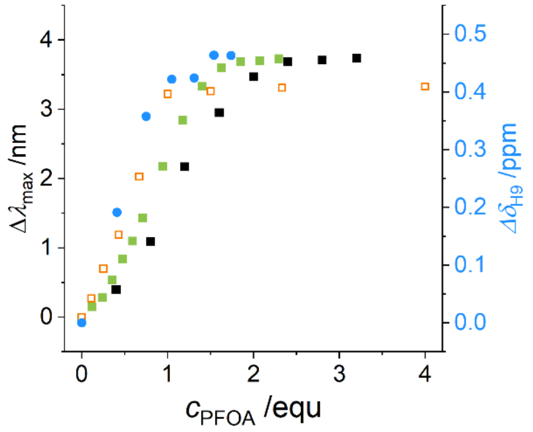

Figure S12. Shifts of the absorption maxima during titrations of **1** with PFOA at concentrations c_1 = 1 µM (black) and 1.8 µM (green). Orange hollow squares denote the Job plot experiments at 2 mM after conversion into a titration plot. The slightly lower endpoint reached for the 2 mM data set (orange hollow squares) is presumably due to the necessity to work at the limits of the linear range of the spectrophotometer even when using microcuvettes, see Section I.c.i. Blue dots correspond to Δ-shift of the guanidine's H9 as derived from the NMR titration (Fig. S16).

VIII. ^{1}H NMR host-guest studies of indicator monomer 1

a)

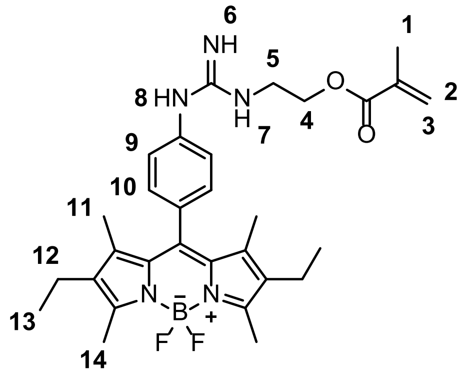

b)

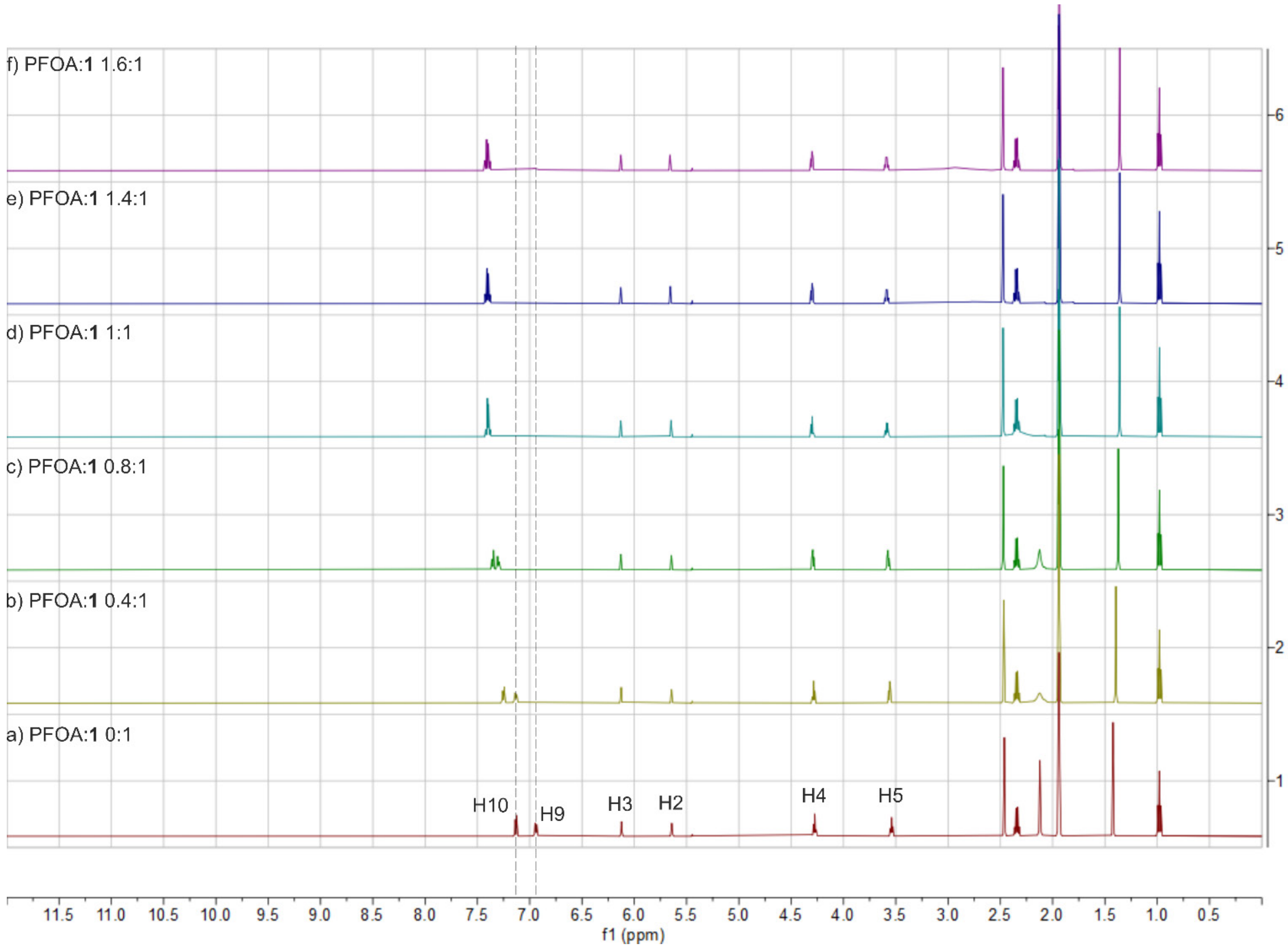

Figure S13. a) Numbering of the assignable protons of **1**; b) ^{1}H NMR (500 MHz, CD_3CN, 300 K) spectra of **1** in the absence (a) and presence of increasing equivalents of PFOA (b–f; 0.4–1.6); c_1 = 5 mM. For shifts of H9 and H10, see Figure S16.

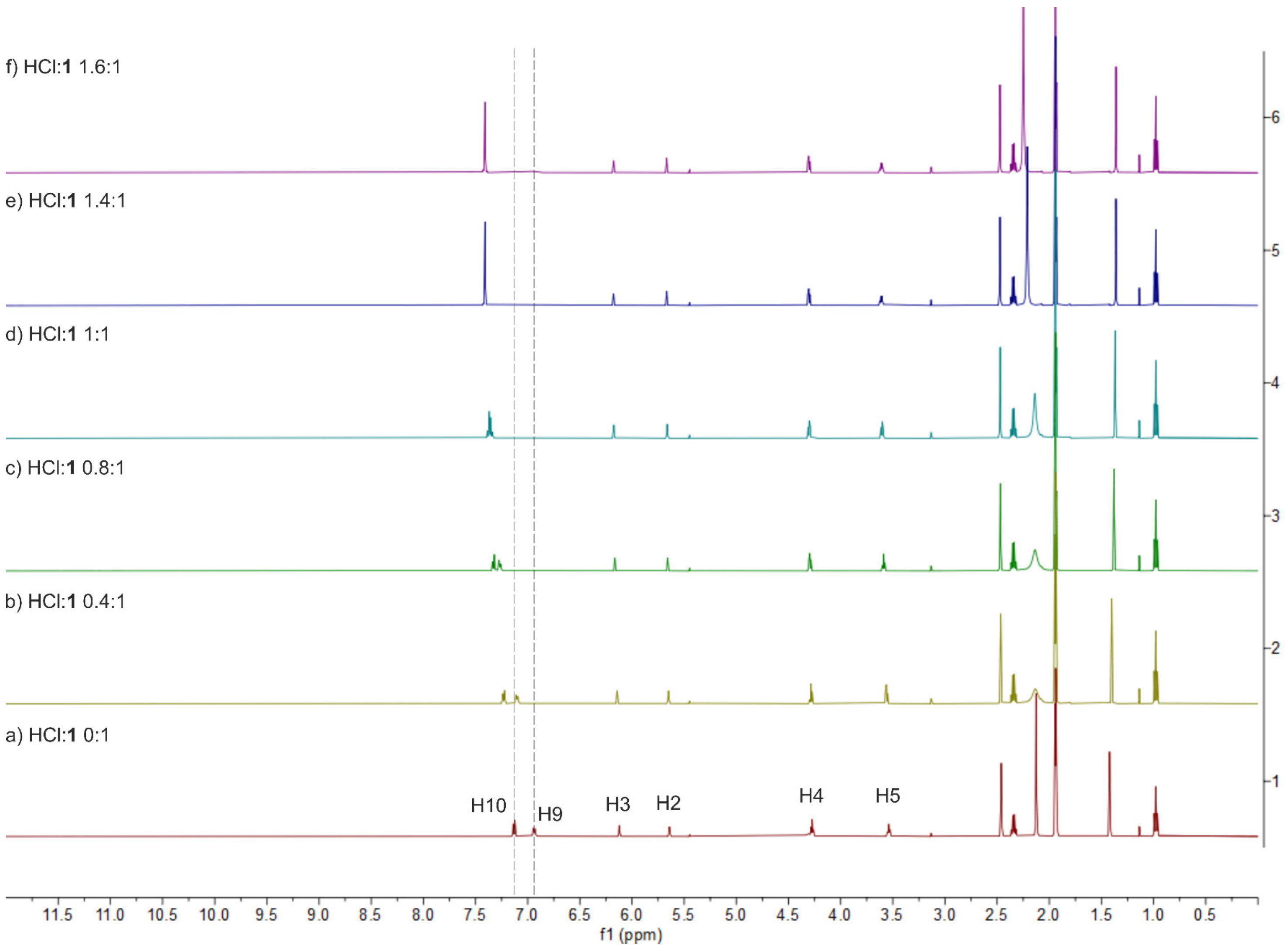

Figure S14. ^{1}H NMR (500 MHz, CD_3CN, 300 K) spectra of **1** in the absence (a) and presence of increasing equivalents of HCl (b–f; 0.4–1.6); c_1 = 5 mM. For shifts of H9 and H10, see Figure S16.

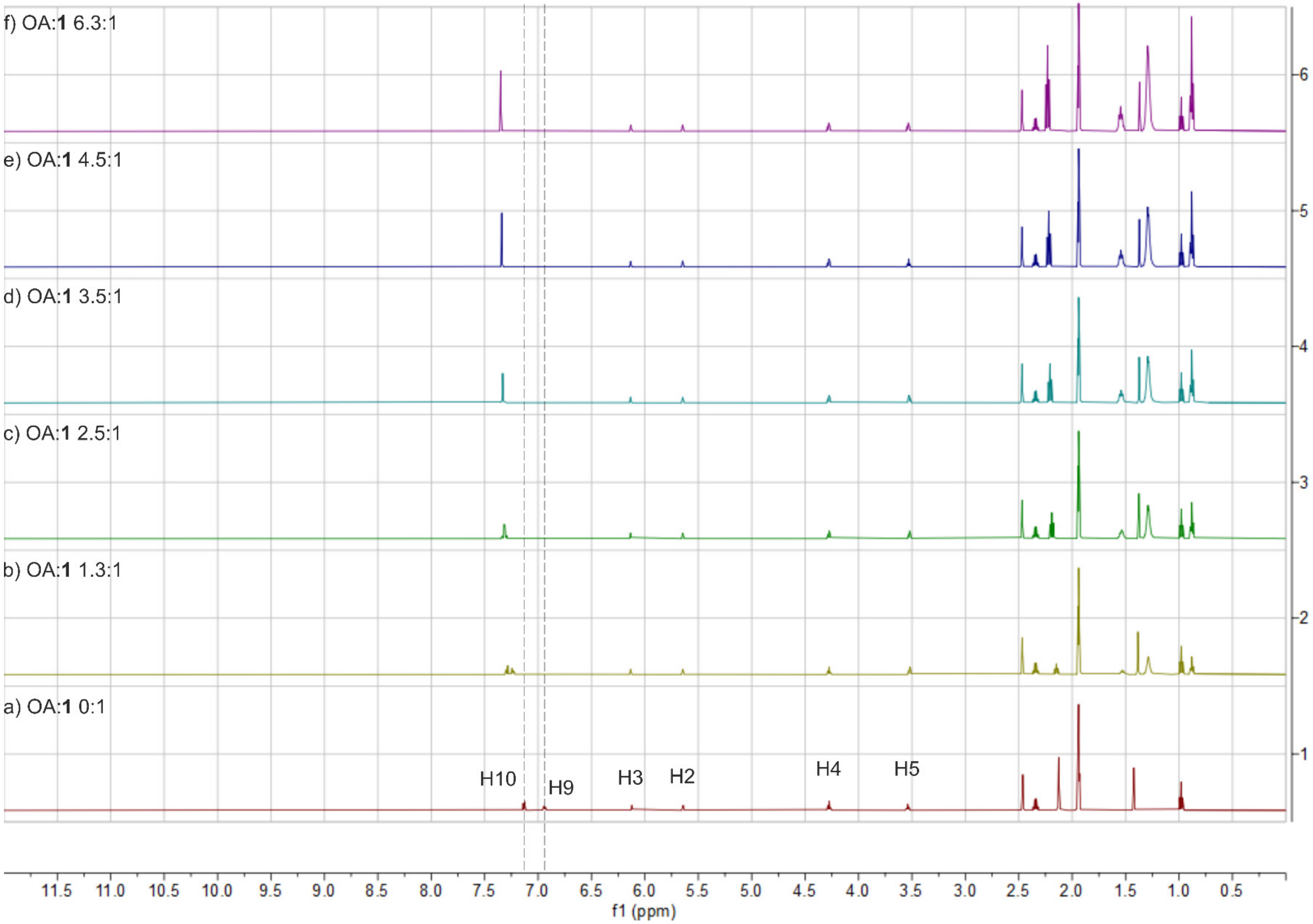

Figure S15. ^{1}H NMR (500 MHz, CD_3CN, 300 K) spectra of **1** in the absence (a) and presence of increasing equivalents of octanoic acid (OA) (b–g; 1.3–6.3); c_1 = 5 mM. For shifts of H9 and H10, see Figure S16.

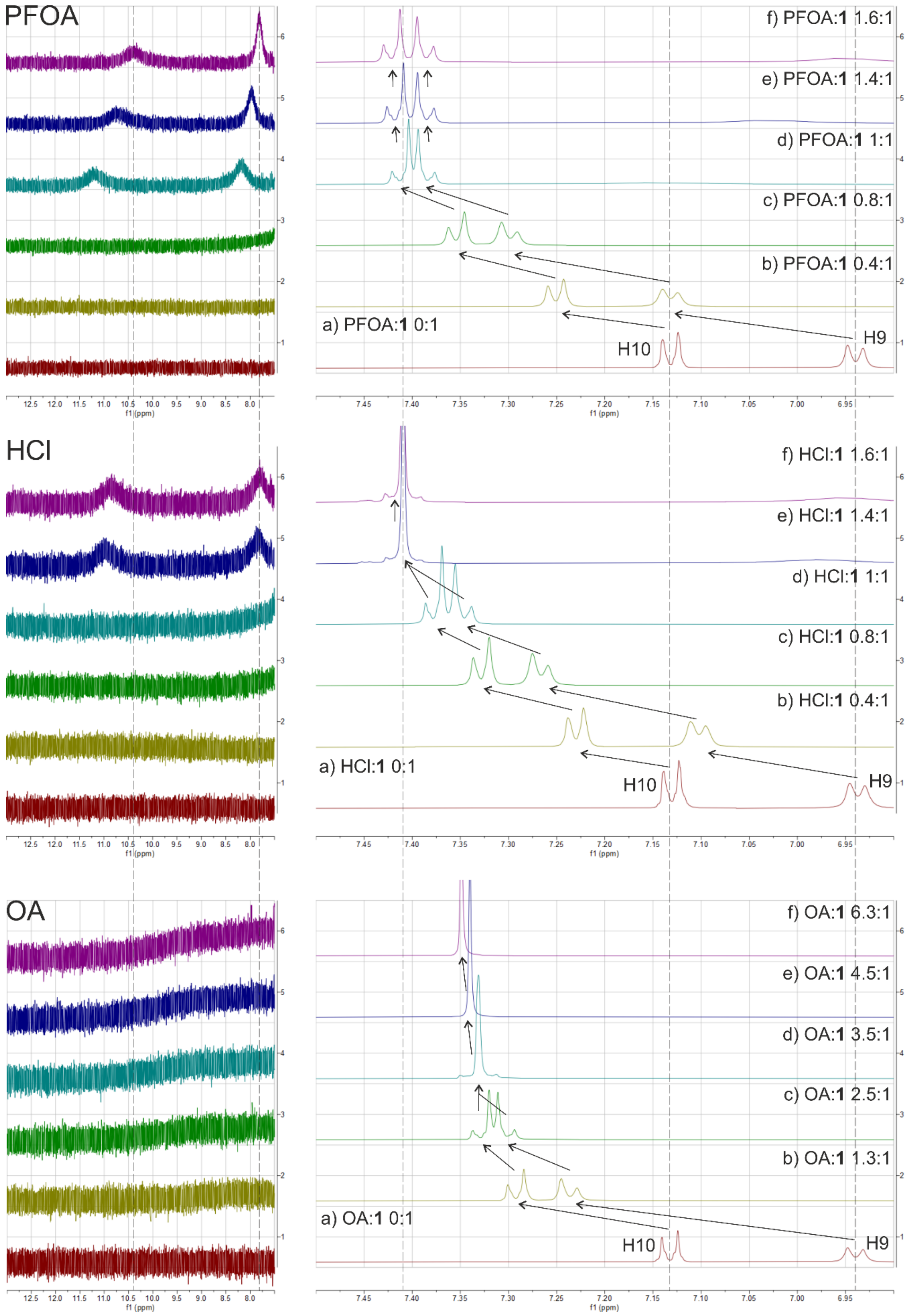

Figure S16. Magnification of the guanidinium proton region (left) and aromatic region (right) of the ^{1}H NMR (500 MHz, CD_3CN, 300 K) titration of **1** with PFOA, HCl and OA shown in Figure S13-S15. The equivalents of analyte of the selected titration steps are given in the plots; c_1 = 5 mM

The most significant shifts during the titration in CD_3CN are the downfield shifts of the aromatic BODIPY protons H9 and H10 for all three analytes, even overlapping for HCl and OA.

Guanidine and guanidinium NH protons exchange rapidly with the solvent and are therefore absent in the first three spectra. Upon increase of PFOA concentration, the guanidinium protons appear at around 8 and 10.5 ppm, indicating that these protons are more fixed and less prone to exchange, which can be attributed to hydrogen bonding between the PFOA's carboxylate moiety and the guanidinium receptor. No guanidinium protons appeared at any point during the titration with OA even at high concentrations indicating no hydrogen bonding. Nonetheless, it can be observed that low guanidinium protons signals appear for HCl at 1.4 equivalents and remain constant upon additional equivalents.

The behaviour of the dye in presence of the three analytes suggests two alternative concerted processes being active: (i) protonation of the guanidine receptor by the acidic guest and (ii.a) strong hydrogen bonding interaction inducing aromatic proton shifts and guanidinium proton locking (case of PFOA) as well as (i) protonation and (ii.b) weak or loose ion-pair formation, mainly inducing shifts of the aromatic protons. For the second type of interaction, (i)/(ii.b), the signals of the guanidinium protons only appear upon addition of a considerable excess of analyte and depend on the analyte's pK_a, i.e., the signals appear for HCl but not for OA.

IX. Sensory particle preparation

*a. Functionalisation of **oSNPs** with APTES (**a@oSNPs**)*

oSNPs (200 mg) were added to anhydrous toluene (40 mL) in a glass vial and sonicated for 30 min. The mixture was magnetically stirred and degassed for 10 min prior to the addition of APTES (94 µL, 2 mmol g^{-1} SiO_2). The reaction was left under stirring and inert atmosphere overnight. After completion of the reaction, the **a@oSNPs** were washed by centrifugation (8965 ×*g*) and redispersion with 4× 15 mL ethanol (96%) to remove any unreacted APTES molecules. The **a@oSNPs** were finally dried overnight in a vacuum at room temperature to obtain an orange solid residue. Ultrasonication was used during the washing steps to remove any physically adsorbed APTES from the particle surfaces.

*b. Functionalisation of **a@oSNPs** with RAFT agent CPCTP (**raft@oSNPs**)*

a@oSNPs (200 mg) were thoroughly mortared and suspended in anhydrous THF (1.2 mL) in a glass vial through ultrasonication for 30 min. In another glass vial with septum, RAFT agent CPCTP (47.5 mg), anhydrous THF (1.7 mL), ethyl chloroformate (16.3 µL) and TEA (23.5 µL) were mixed and degassed for 30 min under stirring and cooling at −78 °C by using a mixture of acetone and liquid nitrogen as the coolant. After ultrasonication, the nanoparticle suspension was shortly degassed and cooled at −78 °C prior to its addition with a syringe to the CPCTP solution. The nanoparticle suspension was rinsed with further anhydrous THF (1 mL), and the resultant mixture degassed for another 5 min still under stirring and cooling at −78 °C. Finally, the reaction mixture was left at room temperature for 24 h under stirring and inert atmosphere. After the completion of the reaction, the **raft@oSNPs** were isolated using hexane (12 mL) and centrifugation (8965 ×*g*); and washed by centrifugation and redispersion with 2× acetone (15 mL) and 2× THF (15 mL) to remove any CPCTP molecules until a clear supernatant was obtained. The **raft@oSNPs** were finally dried overnight in a vacuum at room temperature to obtain an orange-pinkish solid residue. Ultrasonication was used during the washing steps to remove any physically adsorbed RAFT CPCTP molecules from the particle surfaces.

*c. Preparation of orange silica nanoparticle core/green molecularly imprinted polymer shell particle probes (**gMIP@oSNP**)*

Table S10. MIP composition[a]

Substance	MW /g mol^{-1}	ρ /g mL^{-1}	n /µmol	c /mM	m /mg	V /µL	Molar ratio	x_i /M-%	ω_i /m-%	c /µmol mg^{-1} SiO_2
**Monomer 1**	549.5	–	2.8	0.89	1.5	–	1	1.6	3.5	0.14
**PFOA**	414.1	–	2.8	0.89	1.1	–	1	1.6	2.6	0.14
**EGDMA**	198.2	1.051	140	44.0	27.2	25.8	50	80.6	62.7	6.9
**PFOEM**	532.2	1.596	24	7.9	12.9	8.1	9	14.2	29.8	1.2
**ABDV**	248.4	–	3.3	–	0.6	–	1.2	1.9	1.4	0.17
**raft@oSNP**	–	–	–	6.5 g L^{-1}	20.0	–	–	–	–	–
**MeCN**	–	–	–	–	–	3080	–	–	–	–

[a] ρ = density, n = amount of substance, x_i = mole fraction, ω_i = mass fraction

X. Characterisation of particles

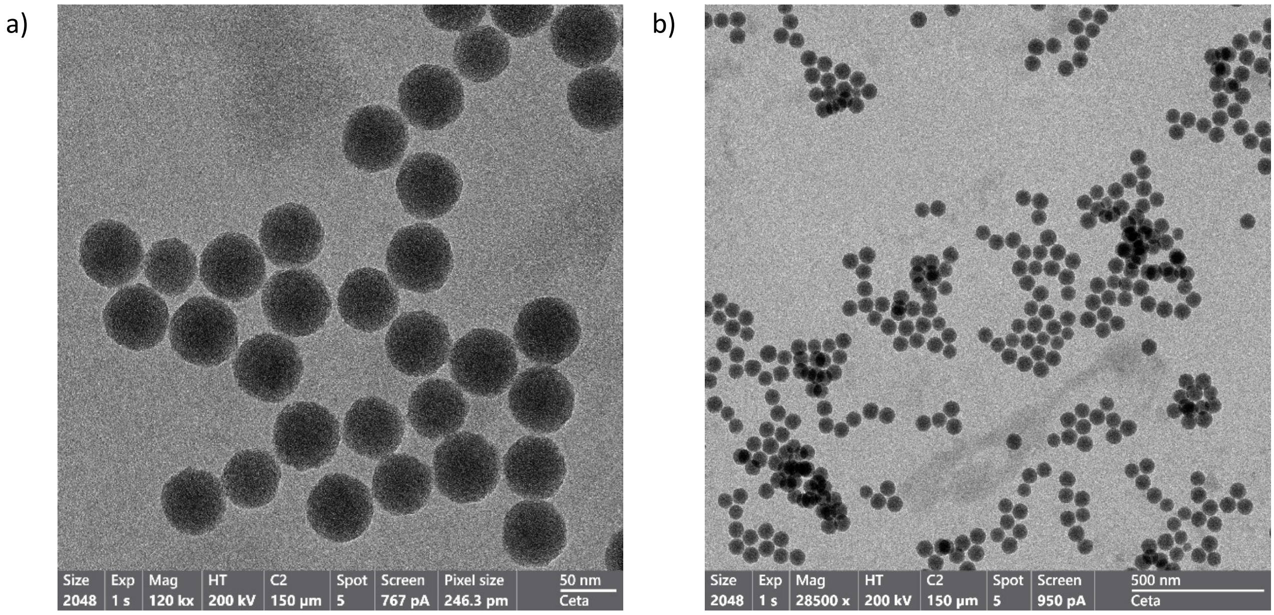

Figure S17. TEM images of the **oSNPs** synthesised by a reverse microemulsion method. Scale bars of 50 nm for a) and 500 nm for b).

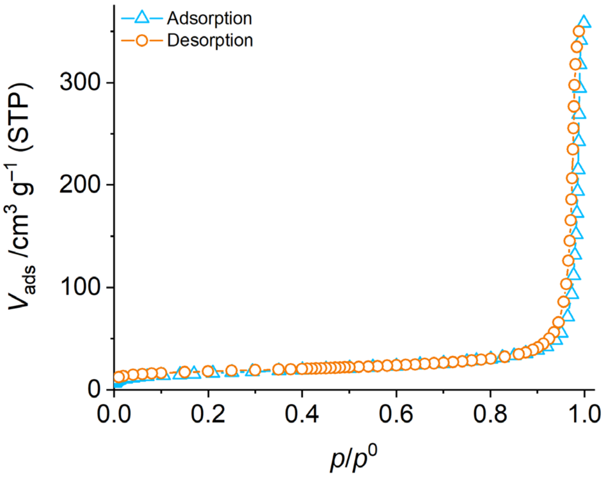

Figure S18. N_2 adsorption/desorption isotherms for **oSNP** (at standard temperature and pressure).

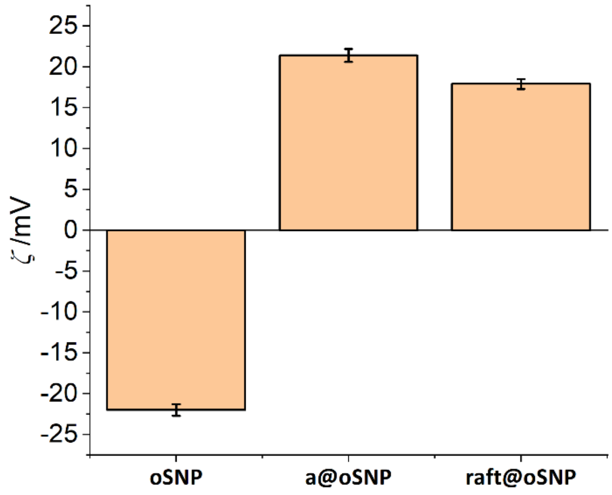

Figure S19. Zeta potential measurements of **oSNP**, **a@oSNP** and **raft@oSNP** at pH = 6. Data are presented as mean ± SD (n = 3 independent experiments).

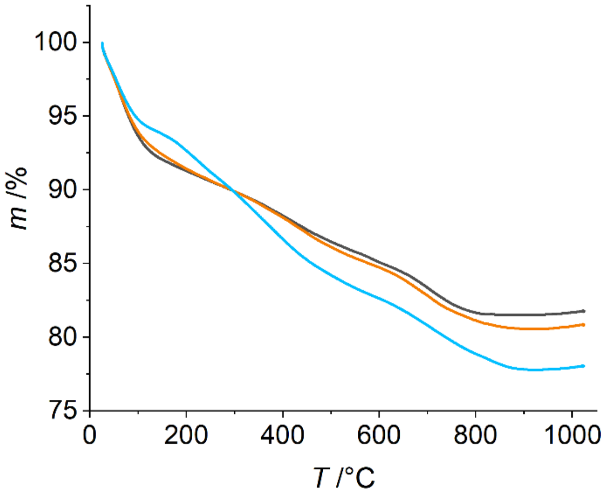

Figure S20. Thermogravimetric analyses of **oSNP** (black), **a@oSNP** (orange) and **raft@oSNP** (blue) with overall mass losses of 18.3%, 19.2% and 22.0%, respectively. TGA analyses were performed from 25 °C to 1000 °C with a heating ramp of 20 °C min^{-1} under synthetic air.

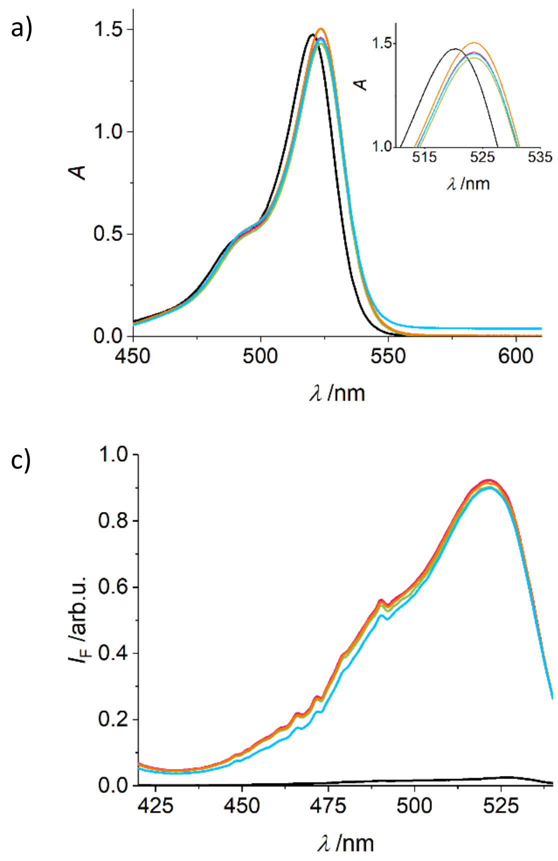

Figure S21. a) Absorption, b) emission (λ_{exc} = 480 nm) and c) excitation (λ_{em} = 550 nm) spectra of **1** (black, 0.9 mM) in MeCN and of **1** (0.9 mM) in the presence of PFOA (red, 0.9 mM), PFOA + EGDMA (green, 44 mM), PFOA + EGDMA + PFOEM (orange, 8 mM) and PFOA + EGDMA + PFOEM + **raft@oSNP** particles (blue, 6.5 g L^{-1}) in MeCN; d) Emission spectra of **1** in presence of all components prior to polymerisation at two different excitation wavelength: 450 (red) and 480 (black) nm. The orange emission of the internal reference of **oSNP** is enhanced when exciting at 450 nm compared to 480 nm.

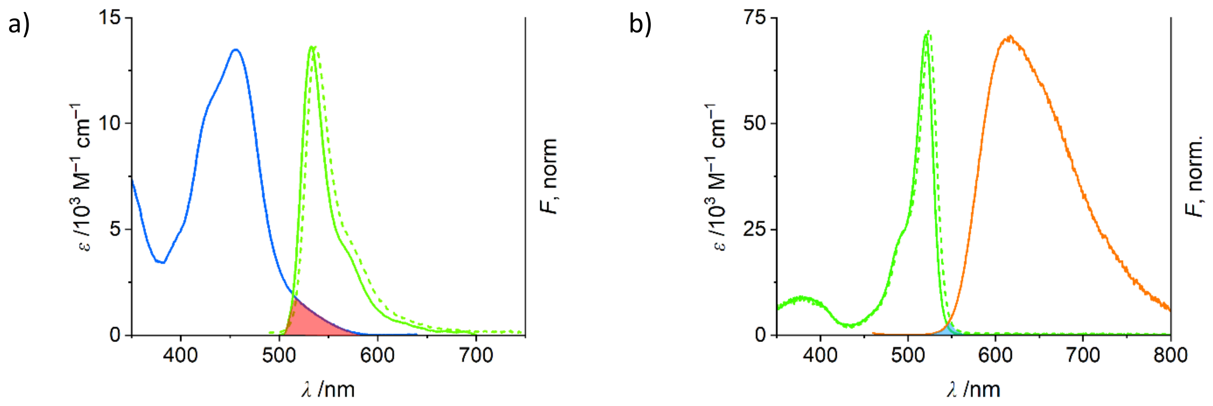

Figure S22. Relevant spectra for the assessment of possible energy transfer interactions between the two dyes of the system. a) Scenario for energy transfer from BODIPY to $Ru(bpy)_3Cl_2$: solid (**1**) and dotted (**1**⊂PFOA) emission spectra (green) of BODIPY donor (in EtOAc) and absorption spectrum (blue) of $Ru(bpy)_3Cl_2$ acceptor (in **oSNP**), spectral overlap marked in red. b) Scenario for energy transfer from $Ru(bpy)_3Cl_2$ to BODIPY: emission spectrum (orange) of $Ru(bpy)_3Cl_2$ donor (in **oSNP**) and solid (**1**) and dotted (**1**⊂PFOA) absorption spectra (green) of BODIPY acceptor (in EtOAc), spectral overlap marked in blue. Colour code corresponding to the band maxima, discussion see text.

Figure S22 shows the spectra of the relevant energy transfer pairs and how small the putative spectral overlap areas are. If all the relevant data of the two chromophores in their respective environments, i.e., EtOAc for **1** and **1**⊂PFOA and silica/**oSNP** for $Ru(bpy)_3Cl_2$, are entered into Equation S14,[44]

$$R_0{}^6 = 8.875 \times 10^{-5} \left(\frac{\kappa^2 \Phi_D J}{n^4}\right) \quad \text{with} \quad J = \int F_D(\lambda)\varepsilon_A(\lambda)\,\lambda^4 d\lambda \qquad \text{Eq. S14}$$

overlap integrals J and energy transfer distances R_0 are calculated as summarised in Table S11. These data suggest that with R_0 between 19–30 Å, the area in which molecules able to participate in energy transfer are to be found on either side of the interface is so small in comparison to the size of the core and the thickness of the shell that energy transfer is unlikely to happen to a degree that can be detectable, if at all.

Table S11. Spectroscopic properties and energy transfer parameters for possible donor–acceptor pairs.

**Donor** ^{a}	**Acceptor** ^{a}	J ^{b} M^{-1} cm^{-1} nm^4	Φ_D ^{c}	n ^{g}	$R_0{}^6$ Å^6	R_0 Å^6
$Ru(bpy)_3Cl_2$ / **oSNP**	**1** / EtOAc	3.39×10^{13}	0.13 ^{d}	1.4237	6.34×10^{7}	20
$Ru(bpy)_3Cl_2$ / **oSNP**	**1**⊂PFOA / EtOAc	7.24×10^{13}	0.13 ^{d}	1.4237	1.36×10^{8}	23
**1** / EtOAc	$Ru(bpy)_3Cl_2$ / **oSNP**	9.37×10^{13}	0.03 ^{e}	1.4237	4.05×10^{7}	19
**1**⊂PFOA / EtOAc	$Ru(bpy)_3Cl_2$ / **oSNP**	8.80×10^{13}	0.54 ^{f}	1.4237	6.84×10^{8}	30

^{a} $Ru(bpy)_3Cl_2$ in **oSNP**, **1** and **1**⊂PFOA in EtOAc. ^{b} J according to Eq. S14. ^{c} Φ_f of the donor. ^{d} Determined against $Ru(bpy)_3Cl_2$ in MeCN, Φ_f = 0.018.[45] ^{e} From Table S4. ^{f} Via enhancement factor at full complexation. ^{g} For simplicity, the medium refractive index of silica NPs[46] and EtOAc[30] was used.

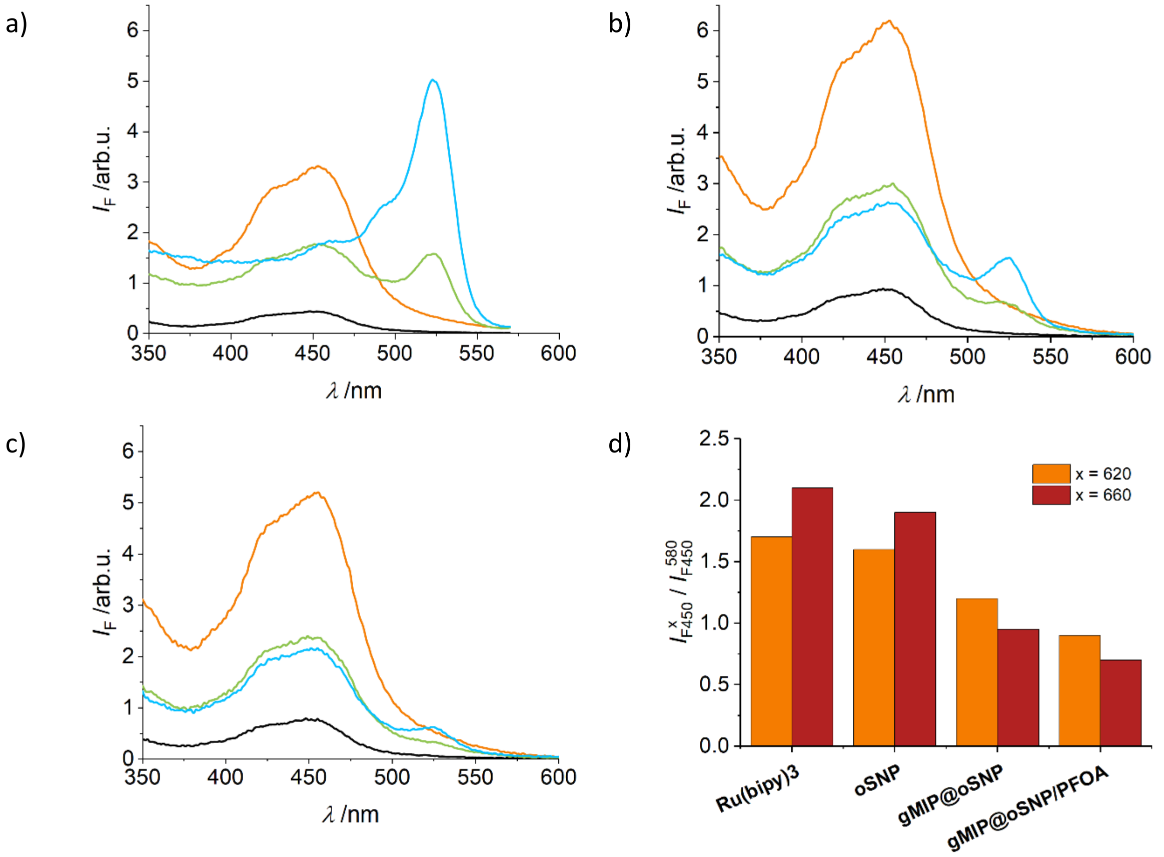

Figure S23. Excitation spectra of $Ru(bpy)_3Cl_2$ (black), **oSNP** (orange), **gMIP@oSNP** (green) and **gMIP@oSNP**/PFOA (blue) with observation wavelengths λ_{obs} = 580 nm (a), 620 nm (b) and 660 nm (c); (d) Intensity ratios at λ_{exc} = 450 nm when observed at the respective λ_{obs} indicated.

The fluorescence excitation spectra were recorded for $Ru(bpy)_3Cl_2$, **oSNP**, **gMIP@oSNP** and **gMIP@oSNP**/PFOA at 580, 620 and 660 nm in MeCN (Figure S23). For all spectra, the $Ru(bpy)_3Cl_2$ band at 453 nm remained mostly unaltered. Potential energy transfer is assessed by comparing the intensity ratio of the excitation spectra at one emission wavelength (620 or 660 nm) against the spectra at a reference wavelength (580 nm) for various systems. If the intensity of the ratio would be larger for the dual fluorescent systems **gMIP@oSNP** and/or **gMIP@oSNP**/PFOA than for the orange dye-only systems ($Ru(bpy)_3Cl_2$, **oSNP**), this would imply that energy is transferred from the shell dye to the core dye. However, the intensity of the ratio is mostly similar in all cases (the slight variation for **gMIP@oSNP** and **gMIP@oSNP**-PFOA due to particle scattering and partial overlap of the two dye excitation bands) and energy transfer processes can be disregarded for this system.

XI. Assays with gMIP@oSNP particle probes

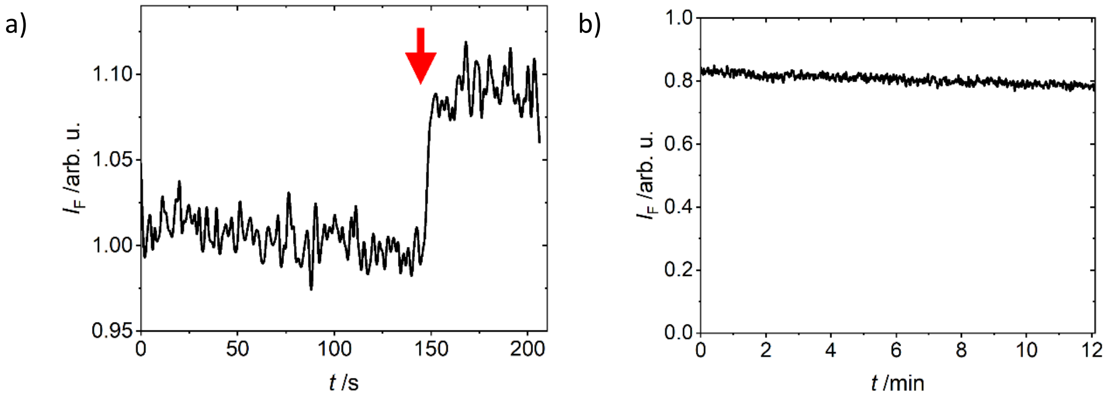

Figure S24. a) Response kinetics of **gMIP@oSNP** upon addition of PFOA (red arrow); b) Emission of **gMIP@oSNP** suspension versus time (MeCN; $c_{\mathbf{gMIP@oSNP}}$ = 62.5 mg L^{-1}; λ_{exc} = 480 nm; λ_{em} = 540 nm).

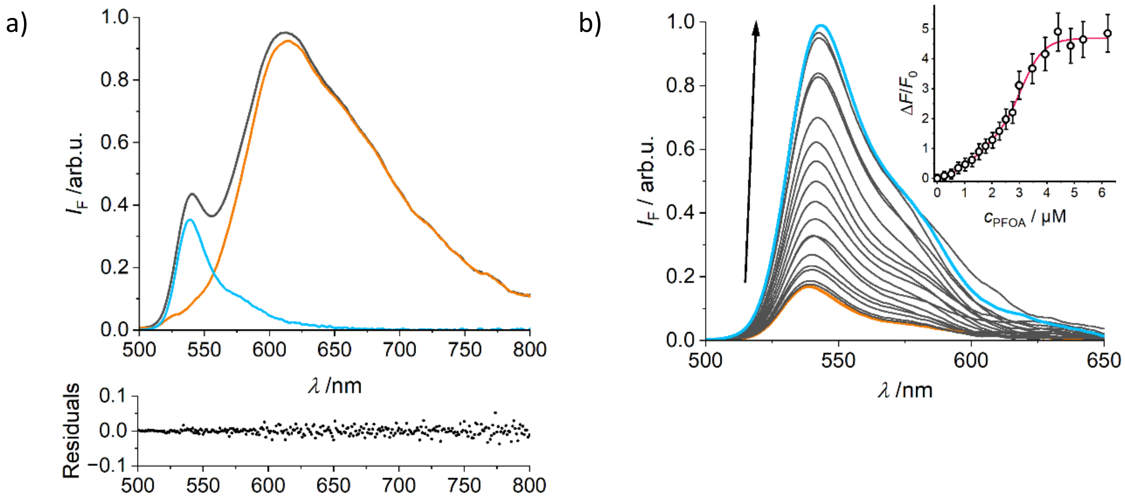

Figure S25. a) Deconvolution of **gMIP@oSNP** emission (black) into contributions from BODIPY indicator **1** in **gMIP** shell (blue) and from Ru(bpy)$_3$Cl$_2$ in **oSNP** core (red) using Microsoft Excel solver; b) Extracted **gMIP** shell signal upon addition of PFOA to a suspension of **gMIP@oSNP** in MeCN ($c_{\mathbf{gMIP@oSNP}}$ = 62.5 mg L^{-1}; λ_{exc} = 480 nm); inset: corresponding concentration-dependent fluorescence change Δ*F*/*F*$_0$. For the inset of b), data are presented as measurement uncertainties, see Eq. S1b (n_r = 3 independent experiments).

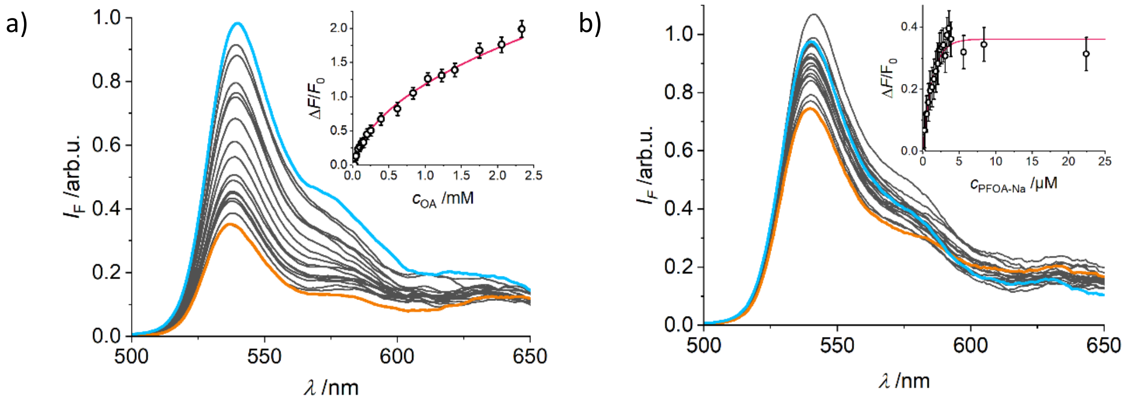

Figure S26. Extracted emission of **gMIP** shell upon increasing concentrations of a) OA and b) PFOA-Na; insets: concentration-dependent fluorescence change Δ*F*/*F*$_0$ (MeCN; $c_{\mathbf{gMIP@oSNP}}$ = 62.5 mg L^{-1}; λ_{exc} = 480 nm). For the insets, data are presented as measurement uncertainties, see Eq. S1b (n_r = 3 independent experiments).

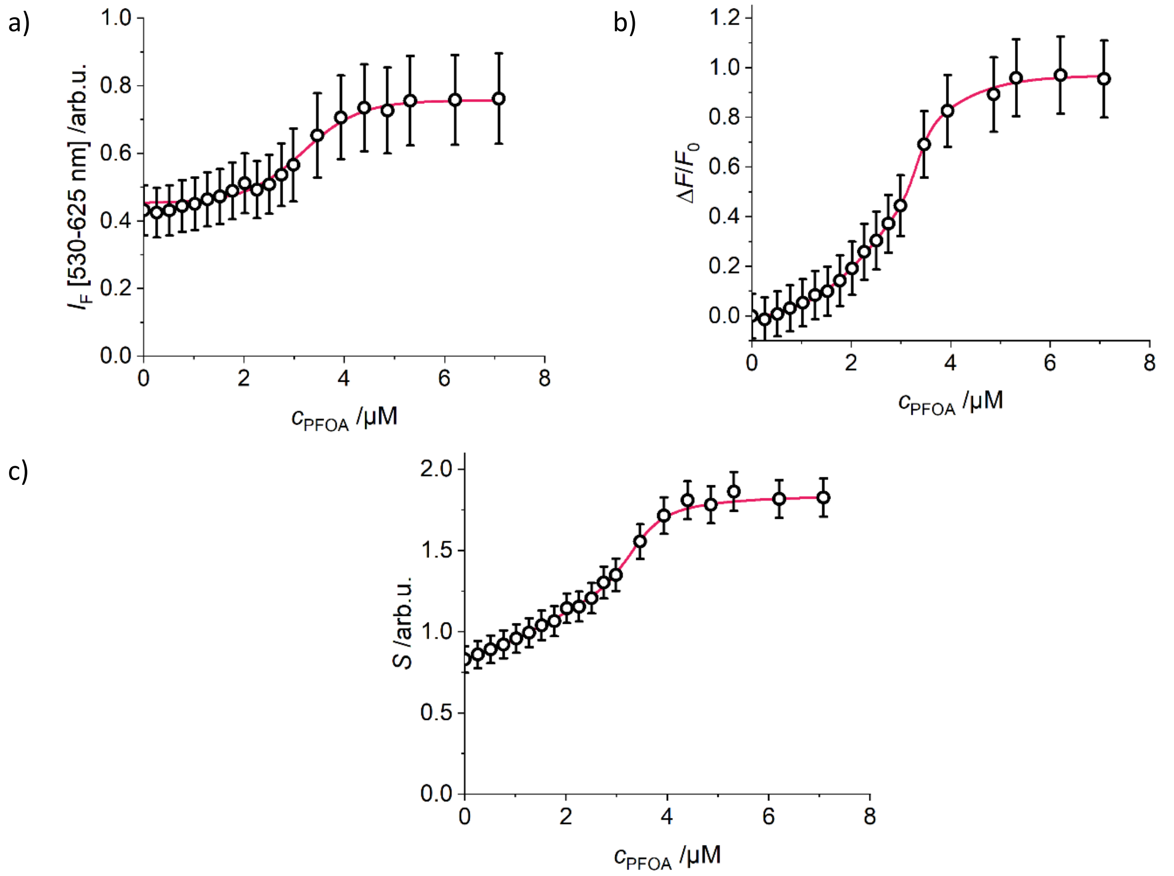

Figure S27. Titration of **gMIP@oSNP** with PFOA: Sigmoidal fitting of a) fluorescence intensity of **gMIP** shell emission (BODIPY emission; average of 3 repeat experiments); b) Reduced fluorescence change $\Delta F/F_0$; c) Shell/core ratiometric fluorescence signal S, see Eq. 5. (Solvent MeCN; $c_{\mathbf{gMIP@oSNP}}$ = 62.5 mg L^{-1}; λ_{exc} = 480 nm). For a), data are presented as measurement uncertainties, see Eq. S1a (n_r = 3 independent experiments). For b) and c), data are presented as measurement uncertainties, see Eq. S1b (n_r = 3 independent experiments).

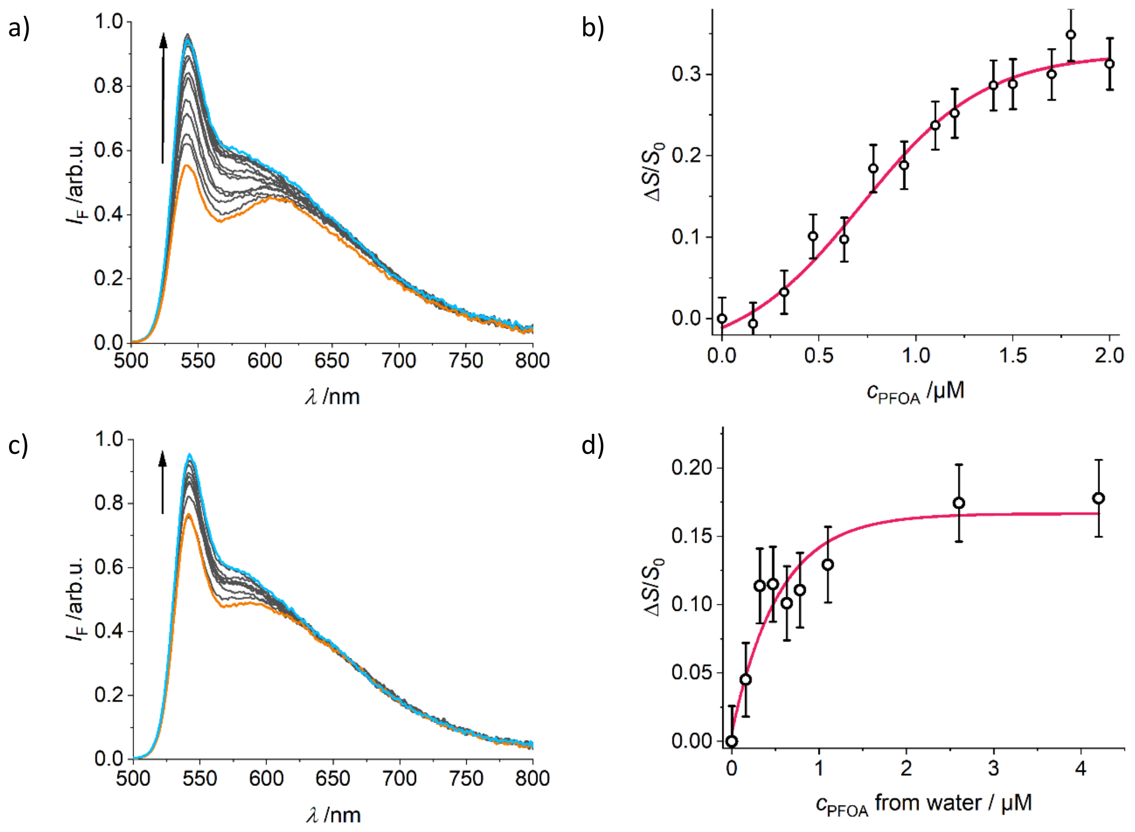

Figure S28. a) Fluorescence titration spectra and b) plot of $\Delta S/S_0$ of **gMIP@oSNP** with increasing concentrations of PFOA in EtOAc; sigmoidal fit in b). Corresponding data obtained for a biphasic extraction assay (PFOA in MilliQ water, **gMIP@oSNP** in EtOAc) in c) and d); $c_{gMIP@oSNP}$ = 62.5 mg L^{-1}; λ_{exc} = 480 nm; sigmoidal fit in d). For b) and d), data are presented as measurement uncertainties, see Eq. S1b (n_r = 3 independent experiments).

Table S12. Binding constants for **1** with PFOA and corresponding saturation values observed for **1** and **gMIP@oSNP** upon addition of PFOA in MeCN, EtOAc and PrOAc.

Solvent	K_S(1+PFOA) /10^5 M^{-1}	max. $\Delta F/F_0$ (1)	max. $\Delta S/S_0$ (gMIP@oSNP)
MeCN	6.7 ± 0.8	39	1.24
EtOAc	1.1 ± 0.1	18	0.35
PrOAc	4.3 ± 0.5	10	0.26

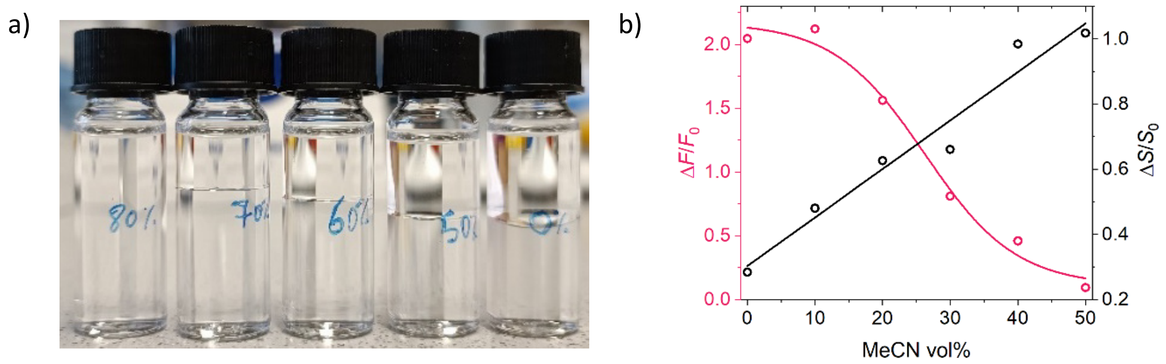

Figure S29. a) Pictures of biphasic ternary MeCN/EtOAc/H_2O systems containing from left to right 80, 70, 60, 50 and 0% MeCN with respect to EtOAc; b) Maximal fluorescence increase $\Delta F/F_0$ for 10 µM of PFOA against vol% of MeCN in EtOAc for dye **1** (red) and the respective $\Delta S/S_0$ for **gMIP@oSNP** (black) in a ternary system (MeCN/EtOAc)/H_2O 1/1 v/v. The EtOAc-water partition coefficient (K_{EW}) has been determined to 6.57 for PFOA in water at pH 7 (data to be published), which corresponds to an extraction efficiency of approx. 85%. To increase this value while minimizing the amount of organic solvent to be used in the assay and converting $PFOA^-$ anions into neutral PFOA, acidification to pH = 2 was carried out, resulting in extraction rates of >95%.

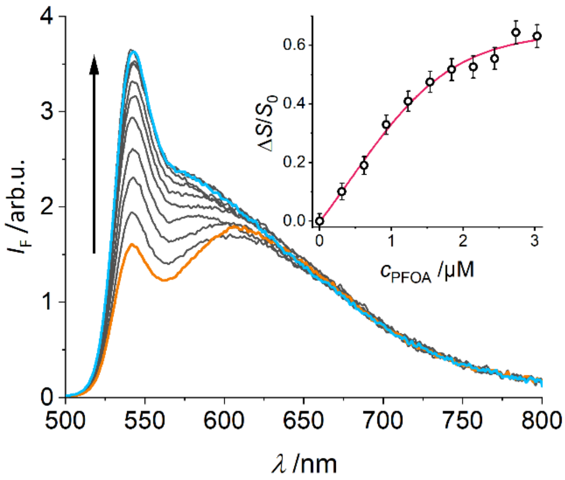

Figure S30. Spectra and (inset) $\Delta S/S$ with sigmoidal fitting of **gMIP@oSNP** response in MeCN/EtOAc to increasing concentrations of PFOA extracted from Milli-Q water ($c_{\mathbf{gMIP@oSNP}}$ = 62.5 mg L^{-1}; λ_{exc} = 480 nm). For the inset, data are presented as measurement uncertainties, see Eq. S1b (n_r = 3 independent experiments).

Table S13. Molecular properties and structural features of analytes and competitors investigated, see Fig. 5b for response behaviour. The meaning of the colour code is described in the text below this table and the chemical structures of the species in side and front view to better visualize entries #3, #7 and #8 are shown in Figure S31.

#	Parameter /Species	PFOA	PFOS	SDBS	SDS	OA	AcOH	AMOX	ENOX
1	logK_{OW} ^{a}	2.69[47]	−1.08 ^{g}[48]	2.0[49]	−2.03[50]	3.05[51]	−0.17[52]	−2.31[53]	−1.02[53]
2	pK_a	1.0 ^{h}[42]	−3.27 ^{i}[54]	−1.34[55]	1.31[56]	4.89[57]	4.75[57]	2.68[58]	6.25[59]
3	$\vartheta_{HG/R}$ ^{b} /°	31.3	72.4	74.8	75.7	31.4	0	79.5	48.7
4	n_{CXm} ^{c}	7	7	16	12	7	1	8	8
5	$\Sigma(\Delta E_{X\text{–}F/H})$ ^{d} /kcal mol^{-1}	−0.47	−0.47	−0.25	−0.25	−0.25	−0.25	−0.25	−0.25
6	$n_{CXm} \times \Sigma(\Delta E_{X\text{–}F})$	−3.29	−3.29	−4.00	−3.00	−1.75	−0.25	−2.00	−2.00
7	l ^{e} /Å	11.08	11.53	20.97	18.49	10.95	3.29	14.56	11.55
8	Shape ^{f}	long cylinder	long cylinder	long cylinder w/ kink	long cylinder	long cylinder	short cylinder	ellipsoid	elliptical disk w/ protrusions

^{a} Octanol-water partition coefficient.

^{b} Tilt angle between plane of Y-shaped acid head group binding motif (COO^- plane) and rest (R) of the molecule, average tilt angle in case of sulphonates/sulphates considering all three potential SOO^- planes; derived from geometry optimized structures.

^{c} Number of CXm units with X = F or H and m = 1, 2 or 3 in the rest (R) of the molecule except for the acid head group.

^{d} Approximation of stabilization of $F_{species}$–F_{PFOEM} and $H_{species}$–F_{PFOEM} interactions upon binding of species into MIP containing PFOEM monomer compared to putative $H_{species}$–$H_{CH\text{-}comonomer}$ interaction, interaction energies taken as $E_{F–F}$ = −0.99 kcal mol^{-1}, $E_{F–H}$ = −0.77 kcal mol^{-1} and $E_{H–H}$ = −0.52 kcal mol^{-1}, calculated for CX_4…CX_4 interactions.[60]

^{e} Length of molecular long axis of geometry optimized molecule (density functional theory (DFT) calculations performed with the B3LYP functional and the 6-311G+(3df,2p) basis set).[22]

^{f} Approximated 3D shape of molecule.

^{g} Calculated value. Otherwise, most works and data bases state that this value cannot be reliably determined for PFOS.

^{h} Diverse values published, most plausible selected.

^{i} Only calculated data available.

The colour code used in the table should help to classify the data. Bright green symbolizes the strongest force in favour of the assay step, bright red the weakest force; the intermediate colours are mixtures of these two colours and correspond to the degree of force that the value of the individual species occupies in the range between the maximum values.

For example, in favour of extraction from water to an organic phase, OA has the highest logK_{OW} of 3.05 (bright green) and AMOX has the lowest logK_{OW} of −2.31 (bright red), leaving SDBS with a logK_{OW} of 2.0 on the green side, yet with a darker green, AcOH with an intermediate logK_{OW} of −0.17 in the brownish region and SDS with a logK_{OW} of −2.03 on the red side, yet with a darker red.

Besides (i) logK_{OW} (entries #1), *monotonic* scales also apply to (ii) pK_a (entries #2), the acid strength being the main driver for converting the guanidine into the guanidinium, (iii) the overall energy gain through interaction of carbon-bound fluorine and hydrogen units in the rest of the molecule with the fluorinated monomer PFOEM in the MIP cavity (entries #6), as the product of the number of CF or CH units n_{CXm} (entries #4) and the energy gain through $F_{species}$–F_{PFOEM} or $H_{species}$–F_{PFOEM} interactions referenced to H–H interactions (entries #5) and (v) the shape of the molecule (entries #8), with a long (straight) cylinder such as the imprinted PFOA being preferred to an ellipsoid or elliptical disk.

The scale for (vi) the tilt angle between acid head group and rest of the molecule, $\vartheta_{HG/R}$ (entries #3), which determines in how far the two Y-shaped hydrogen bonding moieties of MIP-bound indicator monomer and guest species can optimally align to generate the strongest possible signal, and (vii) the length of the molecule (entries #7) have a *maximum* for the imprinted PFOA (bright green) and go into the red regions for more tilted (e.g., AMOX) or longer (e.g., SDBS) as well as less tilted (e.g., AcOH) or shorter (e.g., AcOH) species.

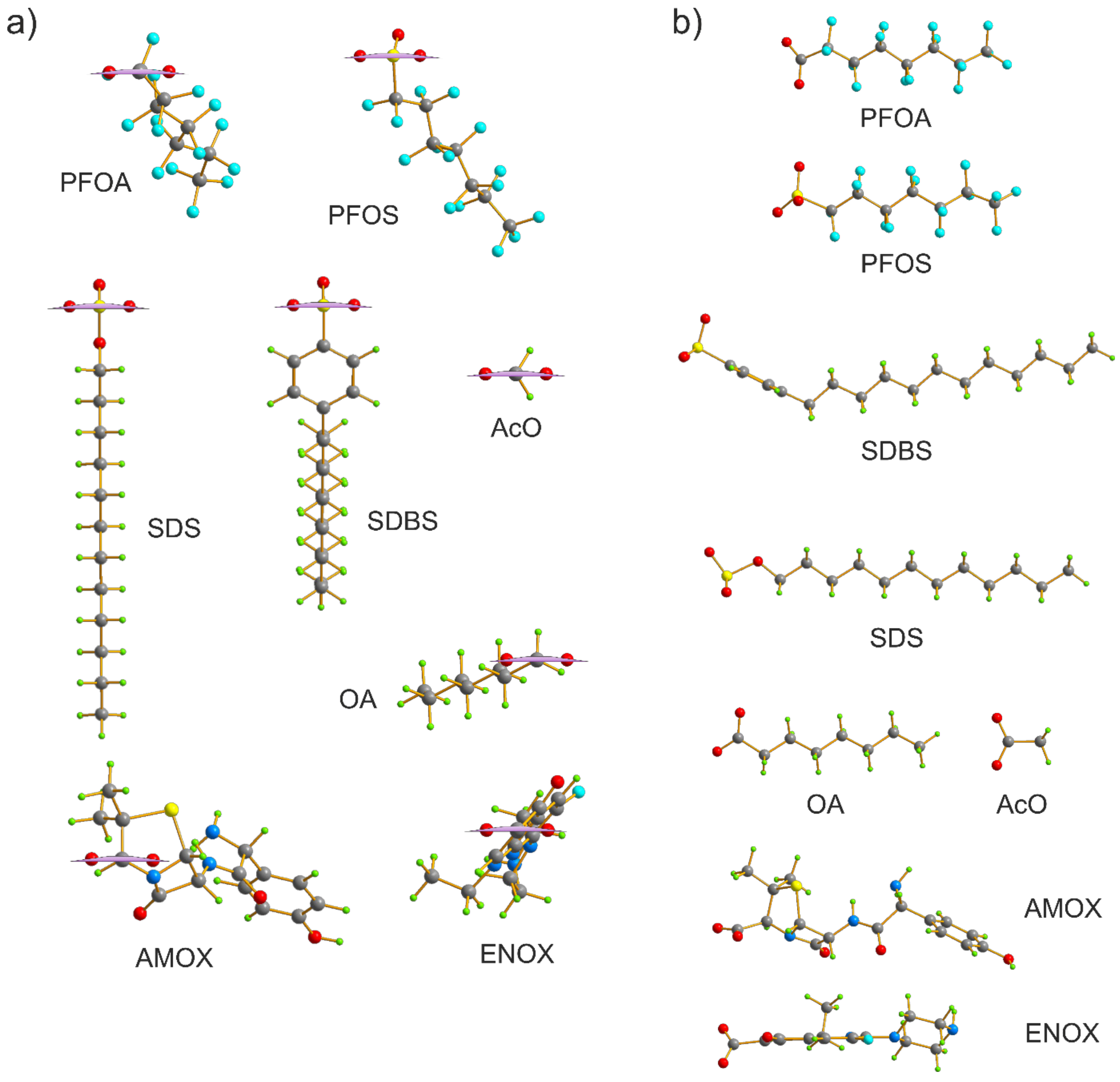

Figure S31. Calculated molecular structures of all species discussed. a) View along the/a planar Y-shaped [O–X–O]⁻ head group moiety (X = C or S) denoted by the magenta plane and b) side view of PFOA, PFOS, SDBS, SDS, OA, AcO, AMOX and ENOX.

XII. Microfluidics

a. Microfluidic platform

In analogy to our earlier work,[61] the system was designed and set up using commercially available low-pressure fittings and transparent perfluoroalkoxyalkane (PFA) tubes (Figure S32). Two inlet tubes (outer diameter OD = 1.6 mm, inner diameter ID = 500 µm) for introducing the PFOA analyte in EtOAc (spiked or obtained from extraction of water samples) and a MeCN suspension of the **gMIP@oSNP** particles (125 mg L^{-1}) were connected to syringes placed on standard syringe pumps (Fusion 100, Chemyx). The pump for the **gMIP@oSNP** syringe was placed vertically to control sedimentation. Both solutions were injected at 11 µL min^{-1} through the inlets of a Topas pearl chain mixing chip (Fluidic 658, microfluidic ChipShop) to ensure mixing of PFOA extract and **gMIP@oSNP** particle suspension (at 62.5 mg L^{-1}) in 7 min. Then, the binary solvent solution went through a detection tube (transparent PFA, OD = 1.6 mm, ID = 800 µm) and fluorescence intensity changes were recorded for 2 min (approx. 240 measured data points) with a modular set up based on a standard opto-mechanical filter cube crossed by the microfluidic tube. The particle probes were excited with two laser diode (LD) modules with automatic power control at 450 (**oSNP**) and 520 nm (**gMIP**) (CW450-05 & CW520-05, Roithner LaserTechnik) powered at 5 V DC. A 40 nm band pass interferential filter (BP 500-40-25, Newport) centred at 500 nm was mounted between the LD at 520 nm and the microfluidic tube. A short pass interferential filter (FLH500, Thorlabs) at 500 nm was mounted between the LD at 450 nm and the microfluidic tube. The fluorescence at 90° was focused with a convex lens (f = 25.4 mm) through a beam splitter (Semrock RazorEdge Beamsplitter 532 RU, AHF analysentechnik) tilted at 45° into a bundle of seven optical fibres (7 x 200 µm core, BFL200HS02, Thorlabs) and into an Ocean Optics spectrometer USB2000+ (t_{int} = 500 ms, background, linearity, dark current and residual light corrections).

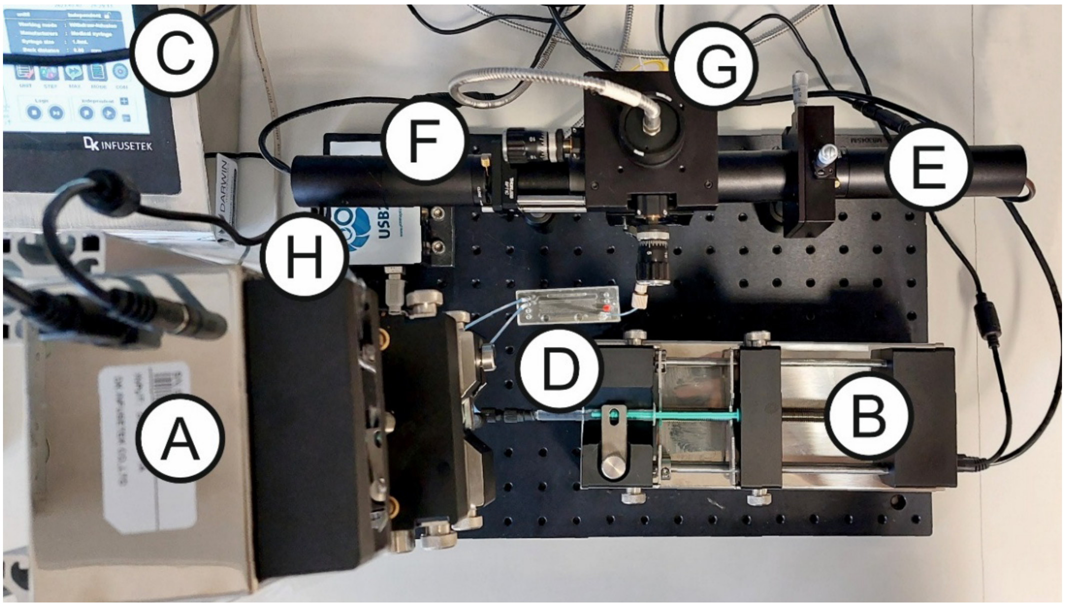

Figure S32. Photograph of the microfluidic setup used in this study: (A) syringe pump in vertical position for injection of the MIP particles; (B) syringe pump for injection of EtOAc extract; (C) controller for syringe pumps; (D) pearl-chain passive mixer chip; (E) **oSNP** excitation channel; (F) **gMIP** excitation channel; (G) emission collection at 90° of the excitation axes of the detection cube with an optical fibre bundle; (H) USB spectrometer. Note that pumps, excitation sources and detectors are commonly used in various projects so that these components are not specifically miniaturized or adapted for the current setup. A final device would naturally look different and more integrated.

b. Mixing efficiency of the passive mixer

The mixing efficiency of the passive mixer (TOPAS Pearl Chain Mixer Chip 658, ChipShop, Jena) was assessed optically. Solutions with a blue and a red dye were each injected through one of the two inlets of the chip and images were taken at the inlet and outlet under different flow regimes. The images' RGB values were extracted, especially the red channel showing the higher contrast between the two dyes. Figure S33 shows the signal measured along the cross-section of the 300 µm wide chip, flowing the red dye in the 0–150 µm (left) section of the chip and the blue dye in the 150–300 µm section. In all cases, the inlet images showed a stable laminar flow with no mixing of the two colours. At the outlet, in all cases (1–40 µL min^{-1}) the passive mixing induced by the pearl chain motifs showed efficient mixing in the chip with homogenous RGB values along the cross section of the channel. Note, that if the extraction step should be integrated into the microfluidic device, extensive optimization procedures as recently described by us in ref. [62] would be required. Here, the device was conceived to guarantee an analysis time of less than 10 min.

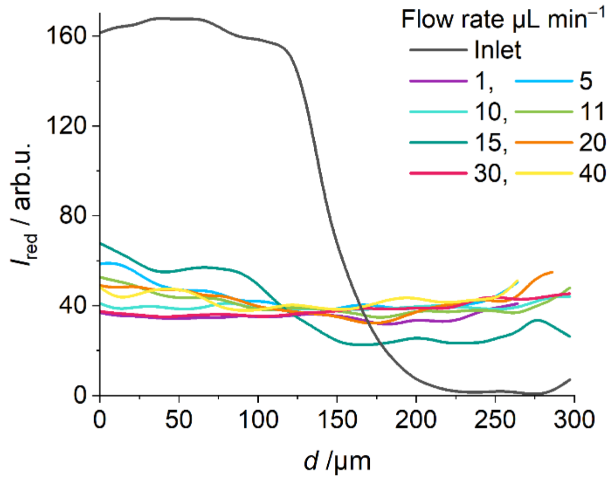

Figure S33. Mixing efficiency of the passive mixer at various flow rates using dyed solutions and measuring the intensity in the red channel across its width *d* at the inlet and outlet of the chip.

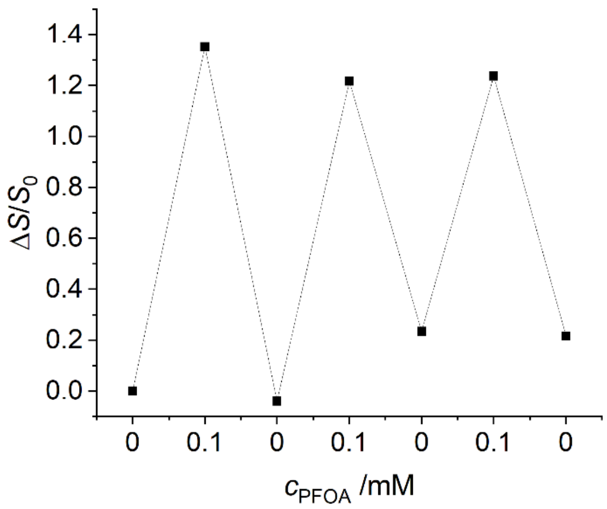

Figure S34. Signal changes upon successively injecting solutions with PFOA concentrations of 0 and 0.1 mM in EtOAc in the microfluidic setup. Also, during the microfluidic titrations, all done in triplicate in a successive manner, no noticeable memory effects were observed.

c. Microfluidic MIPs titration protocol

Prior to microfluidic titration, the PFOA analyte was extracted from 8 mL aqueous sample (Milli-Q water or surface water from Teltow Canal (52°25'32.5"N, 13°32'15.9"E, Berlin, Germany), acidified to pH 2 with HCl, by liquid-liquid extraction with EtOAc (4 mL) as described in Section I.c.iii. Separately, a suspension of 125 mg L^{-1} **gMIP@oSNP** particles in MeCN was prepared. Plastic syringes containing the analyte solution and the particle suspension were then positioned on standard syringe pumps, the injection rate for both pumps was set to 11 µL min^{-1} (final **gMIP@oSNP** concentration of 62.5 mg L^{-1}), and the flows were allowed to equilibrate for 7.5 min before measuring the signals. When solely EtOAc and MeCN were circulating through the tube system, there was no detectable fluorescence. Similarly, no memory effects upon successive injection of PFOA samples was observed.

XIII. Comparison of selected fluorescence-based detection methods for PFAA from literature

Table S14. Selected fluorescence-based detection methods for PFAAs from water[a)]

#	Analyte(s) (interferents)[b)]	Materials	Detection	Instrumentation	Mechanism	LOD model sample[c)]	Range	Assay time (model sample)	LOD realistic sample[d)]	Sample treatment[e)]	Onsite capability	Year	Ref.
1	PFOA, PFHpA, PFHxS (none: short-chain PFCAs, PFOS)	Fluorescein-labelled ssDNA aptamer	Fluorescence	Microwell plate, plate reader	Quencher strand displacement	0.17 µM	5–50 µM	36 + 40 min. conditioning + incubation	5 µM	40 min reaction	–	2022	[63]
2	PFOS, PFOA (none: other PFCAs, PFBS, SDBS, SDS, PFO, HFB)	Erythrosine B + CTAB	Fluorescence	Cuvette, spectrometer	Fluorescence increase by modulation of CTAB micelles in ternary system dye/CTAB/PFAS	12.8 nM (PFOS), 11.8 nM (PFOA)	0.05–10 µM	>20 min. vortexing + incubation	–	n/a	–	2018	[64]
3	PFOS (some: SDS, SDBS; none: CTAB)	HPTS + chitosan	Fluorescence	Cuvette, spectrometer	Fluorescence increase upon HPTS displacement from chitosan	1 nM	0.05–2 µM	25 min. liquid handling + incubation	0.5 µM	Thermal, multi-step, filtration	–	2020	[65]
4	PFOA, PFOS (none tested)	Combinations of fluorinated polymers + fluorinated dyes	Fluorescence	Film or cuvette, spectrometer	Modulation of FRET between polymer and dye through displacement by analyte	140 nM	140 nM–2.5 µM	10 min + 1 h incubation	140 nM	1h incubation	–	2023	[66]
5	PFOS (none: PFCAs, PFBS)	CNDs and berberine chloride hydrate	Fluorescence	Cuvette, spectrometer	Fluorescence increase upon berberine displacement from CNDs	21.7 nM	0.22–50.0 µM	15 min + 10 min incubation	5 µM	Thermal, multi-step, filtration	–	2019	[67]
6	PFOA, PFOS (some: CTAB, OSA, OA)	Guanidinocalix[5]-arene + fluorescein	Fluorescence	Cuvette, spectrometer	Fluorescence increase upon fluorescein displacement from the guanidinocalixarene (IDA)	21.4 nM PFOS, 26.4 nM PFOA	0–0.6 µM PFOA, PFOS	>15 min	31 / 121 nM PFOS, 39 / 120 nM PFOA	SPE	(+)	2019	[68]
7	PFOS, PFCAs (none tested)	DCL of macrocycles from four aromatic dithiols via disulphide linkages, encapsulating LCG fluorophore	Fluorescence, PCA for cluster analysis	Microwell plate, plate reader	Fluorescence increase upon LCG displacement from macrocycles (IDA)	1 nM	–	10 min + 1 h incubation, 5 d (DCL equilibration)	–	n/a	–	2023	[69]
8	PFOA, PFOS, PFHxS (none: SDS)	hLFABP + ANS	Fluorescence	Microwell plate, plate reader	Fluorescence increase upon ANS displacement from hLFABP (IDA)	0.27 µM for PFOA	0.2–300 µM for PFOA	5 min + 5 min, incubation	–	5 min + 5 min, incubation	(+)	2021	[70]
9	PFOA, PFOS (strong: SDBS, 6:2FTS)	AIE luminogen (tetraphenyl-ecene) in water/ acetone in chips	Fluorescence	glass chip, fluorescence microscope	Turn on emission of AIEgen upon PFAS CMC reached (micelles trap AIEgen and prompt aggregation)	0.38 nM	0.1–100 µM	5 min	–	n/a	–	2019	[71]

#	Analyte(s) (interferents)[b)]	Materials	Detection	Instrumentation	Mechanism	LOD model sample[c)]	Range	Assay time (model sample)	LOD realistic sample[d)]	Sample treatment[e)]	Onsite capability	Year	Ref.
10	PFOA (weak: FA, OA, PA, short-chain alcohols and PFCAs)	MPA-decorated CdS QDs	Fluorescence	Cuvette, spectrometer	Fluorescence quenching by QD aggregation	0.3 µM	0.5–40 µM	15 min	10 µM	Textile samples spiked, washed, analysed	–	2015	[72]
11	PFAS (none tested)	Perfluorooctyltri-ethoxysilane functionalized lanthanide-doped tricolour UCNPs	Fluorescence, PCA for cluster analysis	Cuvette, spectrometer, 980 nm laser	Collisional quenching of up-conversion fluorescence	40 nM	0–600 nM	10 min, 30–60 min for real samples	–	30 min, including filtering	–	2020	[73]
12	PFOA, PFOS, PFHxA, PFHpA, PFNA, PFDA (none tested)	Zr porphyrinic luminescent MOF	Fluorescence, PCA for cluster analysis	Microwell plate, plate reader	Static quenching upon PFAS adsorption	0.11 µM for PFOA	0.24–4.8 µM for PFOA	>5 min	5 µM	>5 min, pipetting, incubation, reading	–	2021	[74]
13	PFOA, PFOS (none tested)	Poly(p-phenylene ethynylene) and polyfluorene backbone AFPs (CPdots)	Fluorescence	Film or cuvette, spectrometer	Protonation-induced fluorescence change of AFP	2, 12 nM (PFOA, PFOS) film, 0.8 nM CPdots)	24–97 nM	10 min + 1 h incubation	–	n/a	(+)	2023	[75]
14	PFOA, PFOS (strong: SDS, SDBS; weak: OA, OSA, BSA, BA, PFBA; PFBS, PEGs, Tween, metal ions, sulphate; none: CTAB, inorganic acids, metal ions)	Conjugated polymer PF-DBT-Im	Fluorescence	Cuvette, spectrometer cuvette, 3D-printed holder, smartphone	Dual fluorescent domains capable of FRET in aggregates, altered by electrostatic binding of analyte	6.12, 14.3 nM (PFOA, PFOS)	–	10 min	–	Thermal, precipitat-ion, filtering	+	2023	[76]
15	PFOA (none tested)	Micelles of cyclometalated Iridium(III) complexes on gold surfaces	Luminescence lifetime	Slide, spectrometer	Modulation of surface-bound micelles of lipophilic Ir(III) emitters by hydrophobic analyte	0.25 µM	0.25–2500 µM	>30 min	>0.25 µM	>30 min incubation	–	2024	[77]
16	PFOA, PFOS (none: inorganic acids, non-fluorinated aliphatic and aromatic carboxylic acids; some: SDBS, SDS)	Guanidine-based fluorescent probe, covalently integrated into MIP layer on core nanoparticles	Fluorescence	Microfluidic sensor	Liquid-liquid extraction to organic phase, concerted hydrogen bond- and ion pairing-induced fluorescence change via direct host-guest binding	0.11 µM	1.2–6.1 µM	9 min + 4 min	1.2 µM	buffering	+		This work

Footnotes on next page

[a] PFHxS = perfluorohexanesulphonic acid, ssDNA = single-stranded DNA, PFO = perfluorooctane, SDBS = sodium dodecylbenzenesulphonate, HFB = 2,2,3,3,4,4,4-heptafluoro-1-butanol, CTAB = cetyltrimethylammonium bromide, HPTS = trisodium-8-hydroxypyrene-1,3,6-trisulphonate, FRET = Förster resonance energy transfer, PFBS = nonafluorobutane 1 sulphonic acid, CND = carbon nanodot, OSA = octanesulphonic acid, OA = octanoic acid, IDA = indicator displacement assay, SPE = solid-phase extraction, DCL = dynamic combinatorial library, LCG = lucigenin, PCA = principal component analysis, hLFABP = Human liver fatty acid binding protein, ANS = 1-anilinonaphthalene-8-sulphonic acid, 6:2FTS = 6:2 fluorotelomer sulphonate, AIE = aggregation induced emission, CMC = critical micelle concentration, FA = formic acid, PA = propanioic acid, MPA = 3-mercaptopropionic acid, QD = quantum dot, UCNP = up-conversion nanoparticle, PFNA = perfluorononanoic acid, PFDA = perfluorodecanoic acid, MOF = metal organic framework, AFP = amplifying fluorescent polymer, CPdot = conjugated polymer dot, BSA = butanesulphonic acid, BA = butanoic acid, PEG = polyethylene glycol, PF-DBT-Im = polyfluorene-1,4-dithienylbenzothiadiazole block-co-polymer with alkylimidazole side chains [b] None / some / weak / strong: no / some / weak / strong interference by the compounds listed as reported in or deducible from the respective article. [c] Commonly MilliQ or deionized/distilled water. [d] Commonly spiked tap, river or lake water. [e] Not reported.

XIV. References

1. Kalyanasundaram K. Photophysics, photochemistry and solar energy conversion with tris(bipyridyl)ruthenium(II) and its analogues. *Coord Chem Rev* **46**, 159–244 (1982).

2. Bourson J, Pouget J, Valeur B. Ion-responsive fluorescent compounds. 4. Effect of cation binding on the photophysical properties of a coumarin linked to monoaza- and diaza-crown ethers. *J Phys Chem* **97**, 4552–4557 (1993).

3. Rurack K, Spieles M. Fluorescence Quantum Yields of a Series of Red and Near-Infrared Dyes Emitting at 600−1000 nm. *Anal Chem* **83**, 1232–1242 (2011).

4. Schneider CA, Rasband WS, Eliceiri KW. NIH Image to ImageJ: 25 years of image analysis. *Nat Methods* **9**, 671–675 (2012).

5. Armbruster DA, Pry T. Limit of blank, limit of detection and limit of quantitation. *Clin Biochem Rev* **29 Suppl 1**, S49–52 (2008).

6. Motulsky H, Christopoulos A. Complex dose-response curves. In: *Fitting Models to Biological Data using Linear and Nonlinear Regression*). GraphPad Software, Inc. (2003).

7. Sun Y, Gawlitza K, Valderrey V, Bhattacharya B, Rurack K. Ratiometric Molecularly Imprinted Particle Probes for Reliable Fluorescence Signaling of Carboxylate-Containing Molecules. *ACS Appl Mater Interfaces* **16**, 49944–49956 (2024).

8. Ziessel R, Bonardi L, Retailleau P, Ulrich G. Isocyanate-, isothiocyanate-, urea-, and thiourea-substituted boron dipyrromethene dyes as fluorescent probes. *J Org Chem* **71**, 3093–3102 (2006).

9. Marfin YS, Merkushev DA, Usoltsev SD, Shipalova MV, Rumyantsev EV. Fluorescent Properties of 8-Substituted BODIPY Dyes: Influence of Solvent Effects. *J Fluoresc* **25**, 1517–1526 (2015).

10. Herbich J, Kapturkiewicz A. Electronic Structure and Molecular Conformation in the Excited Charge Transfer Singlet States of 9-Acridyl and Other Aryl Derivatives of Aromatic Amines. *J Am Chem Soc* **120**, 1014–1029 (1998).

11. Bertie JE, Lan Z. Liquid Water−Acetonitrile Mixtures at 25 °C: The Hydrogen-Bonded Structure Studied through Infrared Absolute Integrated Absorption Intensities. *J Phys Chem B* **101**, 4111–4119 (1997).

12. Gagliardi LG, Castells CB, Ràfols C, Rosés M, Bosch E. Static Dielectric Constants of Acetonitrile/Water Mixtures at Different Temperatures and Debye−Hückel A and a_0B Parameters for Activity Coefficients. *J Chem Eng Data* **52**, 1103–1107 (2007).

13. Haynes WM, Lide DR, Bruno TJ. *CRC Handbook of Chemistry and Physics*, 97 edn. CRC Press (2017).

14. Würth C, Grabolle M, Pauli J, Spieles M, Resch-Genger U. Relative and absolute determination of fluorescence quantum yields of transparent samples. *Nat Protoc* **8**, 1535–1550 (2013).

15. Kawai S. [Discussion on decomposition of chloroform]. *Yakugaku Zasshi (J Pharm Soc Jpn)* **86**, 1125–1132 (1966).

16. Nakano K, Konishi T, Imamura Y. Estimation of maximum absorption wavelength of polymethine dyes in visible and near-infrared region based on time-dependent density functional theory. *Chem Phys* **518**, 15–24 (2019).

17. Fabian J. TDDFT-calculations of Vis/NIR absorbing compounds. *Dyes Pigm* **84**, 36–53 (2010).

18. Matulis VE, Ragoyja EG, Ivashkevich OA. Accurate theoretical prediction of optical properties of BODIPY dyes. *Int J Quantum Chem* **120**, e26159 (2020).

19. Le Guennic B, Jacquemin D. Taking Up the Cyanine Challenge with Quantum Tools. *Acc Chem Res* **48**, 530–537 (2015).

20. Kachkovsky OD, Naumenko AP, Borisyuk VI, Obernikhina NV, Slominskiy YL. Nature of Lowest Electron Transitions in Anionic Polymethine Dyes with Keto-Containing Terminal Groups. *Nanosistemi Nanomater Nanotehnologii* **20**, 473–486 (2022).

21. Chibani S, Laurent AD, Le Guennic B, Jacquemin D. Improving the Accuracy of Excited-State Simulations of BODIPY and Aza-BODIPY Dyes with a Joint SOS-CIS(D) and TD-DFT Approach. *J Chem Theory Comput* **10**, 4574–4582 (2014).

22. Frisch MJ*, et al. Gaussian 16 Rev. B.01*. Gaussian, Inc. (2016).

23. Dennington II RD, Keith TA, Millam JM. *GaussView 6.0.16*. Semichem, Inc. (2016).

24. Descalzo AB, Ashokkumar P, Shen Z, Rurack K. On the Aggregation Behaviour and Spectroscopic Properties of Alkylated and Annelated Boron-Dipyrromethene (BODIPY) Dyes in Aqueous Solution. *ChemPhotoChem* **4**, 120–131 (2020).

25. Descalzo AB, Xu HJ, Shen Z, Rurack K. Influence of the meso-substituent on strongly red emitting phenanthrene-fused boron-dipyrromethene (BODIPY) fluorophores with a propeller-like conformation. *J Photochem Photobiol, A* **352**, 98–105 (2018).

26. Kollmannsberger M, Rurack K, Resch-Genger U, Daub J. Ultrafast Charge Transfer in Amino-Substituted Boron Dipyrromethene Dyes and Its Inhibition by Cation Complexation: A New

Design Concept for Highly Sensitive Fluorescent Probes. *J Phys Chem A* **102**, 10211–10220 (1998).

27. Gotor R, Ashokkumar P, Hech M, Keil K, Rurack K. Optical pH Sensor Covering the Range from pH 0-14 Compatible with Mobile-Device Readout and Based on a Set of Rationally Designed Indicator Dyes. *Anal Chem* **89**, 8437–8444 (2017).

28. Karelson MM, Zerner MC. Theoretical treatment of solvent effects on electronic spectroscopy. *J Phys Chem* **96**, 6949–6957 (1992).

29. Maus M, Rettig W, Bonafoux D, Lapouyade R. Photoinduced Intramolecular Charge Transfer in a Series of Differently Twisted Donor−Acceptor Biphenyls As Revealed by Fluorescence. *J Phys Chem A* **103**, 3388–3401 (1999).

30. Reichardt C, Welton T. *Solvents and Solvent Effects in Organic Chemistry*. Wiley-VCH (2011).

31. Connolly ML. Computation of Molecular Volume. *J Am Chem Soc* **107**, 1118–1124 (1985).

32. Lippert E. Spektroskopische Bestimmung des Dipolmomentes aromatischer Vebindungen im ersten angeregten Singulettzustand. *Z Elektrochem* **61**, 962–975 (1957).

33. Izutsu K. *Electrochemistry in Nonaqueous Solutions*, 2 edn. Wiley-VCH (2009).

34. Della Védova CO, Romano RM, Stammler H-G, Mitzel NW. Perfluoropropionic Acid ($CF_3CF_2C(O)OH$): Three Conformations and Dimer Formation. *Molecules* **30**, 1887 (2025).

35. Schneiders AL*, et al.* Structural Characterization of Dimeric Perfluoroalkyl Carboxylic Acid Using Experimental and Theoretical Ion Mobility Spectrometry Analyses. *J Am Soc Mass Spectrom* **36**, 850–861 (2025).

36. Vosburgh WC, Cooper GR. Complex ions. I. The identification of complex ions in solution by spectrophotometric measurements. *J Am Chem Soc* **63**, 437–442 (1941).

37. Rurack K, Radeglia R. Transition metal ion complexes of 2,2 '-bipyridyl-3,3 '-diol and 2,2 '-bipyridyl-3-ol: Spectroscopic properties and solvent-dependent binding modes. *Eur J Inorg Chem*, 2271–2282 (2000).

38. Brace NO. Long Chain Alkanoic and Alkenoic Acids with Perfluoroalkyl Terminal Segments1. *J Org Chem* **27**, 4491–4498 (1962).

39. Burns DC, Ellis DA, Li H, McMurdo CJ, Webster E. Experimental pKa Determination for Perfluorooctanoic Acid (PFOA) and the Potential Impact of pKa Concentration Dependence on Laboratory-Measured Partitioning Phenomena and Environmental Modeling. *Environ Sci Technol* **42**, 9283–9288 (2008).

40. Cheng J, Psillakis E, Hoffmann MR, Colussi AJ. Acid Dissociation versus Molecular Association of Perfluoroalkyl Oxoacids: Environmental Implications. *J Phys Chem A* **113**, 8152–8156 (2009).

41. Goss K-U. The pK_a Values of PFOA and Other Highly Fluorinated Carboxylic Acids. *Environ Sci Technol* **42**, 456–458 (2008).

42. Igarashi S, Yotsuyanagi T. Homogeneous liquid-liquid extraction by pH dependent phase separation with a fluorocarbon ionic surfactant and its application to the preconcentration of porphyrin compounds. *Microchim Acta* **106**, 37–44 (1992).

43. Mukerjee P, Korematsu K, Okawauchi M, Sugihara G. Effect of temperature on the electrical conductivity and the thermodynamics of micelle formation of sodium perfluorooctanoate. *J Phys Chem* **89**, 5308–5312 (1985).

44. Wu PG, Brand L. Resonance Energy Transfer: Methods and Applications. *Anal Biochem* **218**, 1–13 (1994).

45. Ishida H, Tobita S, Hasegawa Y, Katoh R, Nozaki K. Recent advances in instrumentation for absolute emission quantum yield measurements. *Coord Chem Rev* **254**, 2449–2458 (2010).

46. Khlebtsov BN, Khanadeev VA, Khlebtsov NG. Determination of the Size, Concentration, and Refractive Index of Silica Nanoparticles from Turbidity Spectra. *Langmuir* **24**, 8964–8970 (2008).

47. ECHA Committees for Risk Assessment and for Socio-economic Analysis. *Background document to the Opinion on the Annex XV dossier proposing restrictions on Perfluorooctanoic acid (PFOA), PFOA salts and PFOA-related substances*, Version 2.0. European Chemicals Agency (ECHA) (2018). https://echa.europa.eu/documents/10162/8059e342-1092-410f-bd85-80118a5526f5

48. Stevens JB, Coryell A. *Surface Water Quality Criterion for Perfluorooctane Sulfonic Acid*. No. STS Project 200604796. Minnesota Pollution Control Agency (2007)

49. European Chemicals Agency (ECHA). *Sodium dodecylbenzenesulfonate*, Brief Profile, 01.05.2024. ECHA (2024). https://echa.europa.eu/de/brief-profile/-/briefprofile/100.042.422

50. European Chemicals Agency (ECHA). *Sodium dodecyl sulphate*, Brief Profile, 18.05.2024. ECHA (2024). https://echa.europa.eu/de/brief-profile/-/briefprofile/100.005.263

51. European Chemicals Agency (ECHA). *Octanoic acid*, Brief Profile, 18.05.2024. ECHA (2024). https://echa.europa.eu/de/brief-profile/-/briefprofile/100.004.253

52. European Chemicals Agency (ECHA). *Acetic acid*, Brief Profile, 21.05.2024. ECHA (2024). https://echa.europa.eu/de/brief-profile/-/briefprofile/100.000.528

53. Verlicchi P, *et al.* Selection of indicator contaminants of emerging concern when reusing reclaimed water for irrigation — A proposed methodology. *Sci Total Environ* **873**, 162359 (2023).

54. Brooke D, Footitt A, Nwaogu TA. *Environmental Risk Evaluation Report: Perfluorooctanesulphonate (PFOS)*. Environment Agency, UK (2004). https://www.gov.uk/government/uploads/system/uploads/attachment_data/file/290857/scho1009brbl-e-e.pdf

55. Australian Industrial Chemicals Introduction Scheme. *Linear alkylbenzene sulfonates. Evaluation statement*. Australian Government (2022)

56. Australian Industrial Chemicals Introduction Scheme. *Lauryl (dodecyl) sulfates. Evaluation statement*. Australian Government (2022)

57. Kortüm G, Vogel W, Andrussow K. Dissociation constants of organic acids in aqueous solution. *Pure Appl Chem* **1**, 187–536 (1960).

58. Homsirikamol C, Sunsandee N, Pancharoen U, Nootong K. Synergistic extraction of amoxicillin from aqueous solution by using binary mixtures of Aliquat 336, D2EHPA and TBP. *Sep Purif Technol* **162**, 30–36 (2016).

59. Lin C-E, Deng Y, Jr., Liao W-S, Sun S-W, Lin W-Y, Chen C-C. Electrophoretic behavior and pK_a determination of quinolones with a piperazinyl substituent by capillary zone electrophoresis. *J Chromatogr A* **1051**, 283–290 (2004).

60. Tsuzuki S, Uchimaru T. Magnitude of attraction in CF_4-CH_4 interactions: Are CF_4-CH_4 interactions weaker than average of CF_4-CF_4 and CH_4-CH_4 interactions? *J Fluorine Chem* **231**, 109468 (2020).

61. Bell J, Climent E, Hecht M, Buurman M, Rurack K. Combining a Droplet-Based Microfluidic Tubing System with Gated Indicator Releasing Nanoparticles for Mercury Trace Detection. *ACS Sens* **1**, 334–338 (2016).

62. Burnage SC, Bell J, Wan W, Kislenko E, Rurack K. Combining a hybrid chip and tube microfluidic system with fluorescent molecularly imprinted polymer (MIP) core–shell particles for the derivatisation, extraction, and detection of peptides with N-terminating phosphorylated tyrosine. *Lab on a Chip* **23**, 466–474 (2023).

63. Park J, Yang K-A, Choi Y, Choe JK. Novel ssDNA aptamer-based fluorescence sensor for perfluorooctanoic acid detection in water. *Environ Int* **158**, 107000 (2022).

64. Cheng Z, Du L, Zhu P, Chen Q, Tan K. An erythrosin B-based “turn on” fluorescent sensor for detecting perfluorooctane sulfonate and perfluorooctanoic acid in environmental water samples. *Spectrochim Acta, Part A* **201**, 281–287 (2018).

65. He J, Su Y, Sun Z, Zhang R, Wu F, Bai Y. A chitosan-mediated “turn-on” strategy for rapid fluorometric detection of perfluorooctane sulfonate. *Microchem J* **157**, 105030 (2020).

66. Concellón A, Swager TM. Detection of Per- and Polyfluoroalkyl Substances (PFAS) by Interrupted Energy Transfer. *Angew Chem, Int Ed* **62**, e202309928 (2023).

67. Cheng Z*, et al.* Highly selective fluorescent visual detection of perfluorooctane sulfonate via blue fluorescent carbon dots and berberine chloride hydrate. *Spectrochim Acta, Part A* **207**, 262–269 (2019).

68. Zheng Z*, et al.* Guanidinocalix[5]arene for sensitive fluorescence detection and magnetic removal of perfluorinated pollutants. *Nat Commun* **10**, 5762 (2019).

69. Harrison EE, Waters ML. Detection and differentiation of per- and polyfluoroalkyl substances (PFAS) in water using a fluorescent imprint-and-report sensor array. *Chem Sci* **14**, 928–936 (2023).

70. Mann MM, Tang JD, Berger BW. Engineering human liver fatty acid binding protein for detection of poly- and perfluoroalkyl substances. *Biotechnol Bioeng* **119**, 513–522 (2022).

71. Fang C, Wu JJ, Sobhani Z, Al Amin M, Tang YH. Aggregated-fluorescent detection of PFAS with a simple chip. *Anal Methods* **11**, 163–170 (2019).

72. Liu Q, Huang AZ, Wang N, Zheng G, Zhu LH. Rapid fluorometric determination of perfluorooctanoic acid by its quenching effect on the fluorescence of quantum dots. *J Lumin* **161**, 374–381 (2015).

73. Yin M, Che L, Jiang S, Deng Q, Wang S. Sensing of perfluorinated compounds using a functionalized tricolor upconversion nanoparticle based fluorescence sensor array. *Environ Sci Nano* **7**, 3036–3046 (2020).

74. Chen B, Yang Z, Qu X, Zheng S, Yin D, Fu H. Screening and Discrimination of Perfluoroalkyl Substances in Aqueous Solution Using a Luminescent Metal–Organic Framework Sensor Array. *ACS Appl Mater Interfaces* **13**, 47706–47716 (2021).

75. Concellón A, Castro-Esteban J, Swager TM. Ultratrace PFAS Detection Using Amplifying Fluorescent Polymers. *J Am Chem Soc* **145**, 11420–11430 (2023).

76. Chen XY*, et al.* Two-in-one platform based on conjugated polymer for ultrasensitive ratiometric detection and efficient removal of perfluoroalkyl substances from environmental water. *Sci Total Environ* **860**, 160467 (2023).

77. Zhang K*, et al.* Luminescence Lifetime-Based Sensing Platform Based on Cyclometalated Iridium(III) Complexes for the Detection of Perfluorooctanoic Acid in Aqueous Samples. *Anal Chem* **96**, 1565–1575 (2024).
